# Intact, recombinant, and spliced forms of endogenous mouse mammary tumor viruses in inbred and wild mice

**DOI:** 10.1128/jvi.00079-25

**Published:** 2025-03-13

**Authors:** Oscar Lam, Esther Shaffer, Guney Boso, Christine A. Kozak

**Affiliations:** 1 Laboratory of Immunoregulation and Infectious Diseases, National Institutes of Health, Bethesda, Maryland, USA; The Ohio State University, Columbus, Ohio, USA

**Keywords:** endogenous retroviruses, mouse mammary tumor viruses, superantigen gene, positive selection, spliced endogenous retroviruses

## Abstract

Endogenous retroviruses (ERVs) are chromosomally integrated viral copies that represent relics of past infections. Analysis of the sequenced genomes of 17 mouse strains, *Mus musculus* subspecies, and *Mus spretus* identified 29 ERVs of mouse mammary tumor viruses (MMTVs), termed *Mtvs*. The 15 laboratory mouse *Mtvs* are each present in multiple strains reflecting their common breeding history; most predate the development of inbred strains and were likely acquired by *Mus musculus domesticus* progenitors but have no orthologs in wild mice, whereas four, including the intact *Mtv1*, were likely endogenized more recently. One of the 14 *Mtvs* found in wild mice was distributed over a broad geographic range in southeast Asia. Most *Mtvs* are full-length, with multiple open reading frames, but *Mtvs* from many wild mice have an unusual envelope deletion corresponding to an intron of the viral *rem* accessory gene, suggesting its derivation from spliced MMTV cDNAs. These deleted *envs* have open reading frames, are found in globally distributed mice, and show subspecies-specific sequence variation consistent with their recurrent generation. The highly variable MMTV *sag* gene, responsible for resistance to exogenous infection, exhibits evidence of recombination as well as positive selection, consistent with its role in antiviral defense. In contrast, the spread of *Mtvs* in *Mus musculus* populations is not marked by an active arms race pitting the MMTV envelope against its cellular receptor. Thus, the acquisition of potentially disease-inducing *Mtvs* is a recent and ongoing process in *Mus* accompanied by recombination, positive selection, and a recurrent envelope deletion.

**IMPORTANCE:**

Endogenous retroviruses (ERVs) are copies of viral genomes inserted into host chromosomes, producing a fossil record of past infections and virus-host co-adaptations. ERVs of mouse mammary tumor viruses (*Mtvs*) were found in all common laboratory strains, all *Mus musculus* subspecies, and a sister species, *Mus spretus*. Most laboratory mouse *Mtvs* predate inbred strain origins and were acquired by *M. musculus domesticus*, but although widely shared among strains, none of these were found in wild mice. Among wild mouse *Mtvs*, only one showed a broad geographic distribution. All *M. musculus* subspecies carry *Mtvs* with a large envelope deletion corresponding to the processed mRNA for the viral *rem* gene; such *Mtvs* likely derive from spliced viral mRNA. The *Mtv sag* gene responsible for resistance to exogenous infection is under purifying selection and has been subject to recombination, whereas the *Mtv* envelope and its cellular receptor show no evidence of genetic conflicts.

## INTRODUCTION

Infectious retroviruses (XRVs) insert DNA copies of their genomes into host cell chromosomes as they replicate. These copies can be passed to subsequent generations, and if germline cells are infected, such insertions can become permanent parts of the host genome termed endogenous retroviruses (ERVs). ERVs represent 8%–10% of mammalian genomes, including those of humans and mice (1, 2). Although ERVs are subject to mutagenesis that disables their coding capacity, and over long periods of time can render them unrecognizable, these fossils can provide a record of past infections, sometimes identifying viruses that are no longer extant and providing a window into the evolutionary history of ERV acquisitions and virus-host co-adaptations (3).

ERVs have been identified for all seven retroviral genera (alpha, beta, delta, gamma, epsilon, lenti, and spuma), although there are major differences in their numbers and distribution among vertebrate species. The gamma and gamma-like ERVs are the most common and show evidence of cross-species transmission (4, 5), whereas lentiretroviral ERVs have been found in a few species, for example (6). The earliest studies on retroviruses focused on pathogenic animal retroviruses (7) and included among these are the mouse mammary tumor viruses (MMTVs). In their natural hosts, these betaretroviruses exist as often pathogenic XRVs and are also found as ERVs, termed *Mtvs*, two of which, *Mtv1* and *Mtv2*, were originally implicated in disease induction (8, 9). Mammary tumors result from milk-borne MMTV viruses transmitted to nursing pups that develop tumors as adults (10, 11).

MMTVs are complex retroviruses with *gag*, *pro* (protease), *pol* (polymerase), and *env* (envelope) genes flanked by long terminal repeats (LTRs), and they also encode three accessory proteins. The *dut-pro* gene produces a dUTPase that limits the incorporation of dUTP into DNA (12, 13). The s*ag* gene-encoded superantigen is a type II transmembrane protein that, in infectious viruses, promotes MMTV infection by inducing T cell proliferation and the ultimate deletion of Sag-reactive T cells (14), whereas *sag* expression from *Mtvs* deletes entire classes of T-cells preventing infection by exogenous MMTVs having the corresponding Sag specificity (15). The *rem* gene product is functionally analogous to the *rev* gene of HIV-1 in its regulation of the nuclear export of unspliced and partially spliced viral mRNAs (16, 17), and Rem has been additionally implicated in the inhibition of activation-induced cytidine deaminase (AID) mutagenesis of MMTV (18).

Early studies reliant on DNA solution hybridization and Southern blots identified *Mtv*s in selected laboratory mice, crudely mapped their chromosomal locations, and determined their distribution in a few common strains of mice with emphasis on mouse strains susceptible to naturally occurring mammary tumors, notably C3H and GR (19–23). These approaches also found MMTV sequences in subspecies of the house mouse, *Mus musculus,* and in the related species, *Mus spretus* (24–27). One full-length *Mtv* was sequenced (*Mtv1*, GenBank number AF228550), and partial sequences, mainly *env* and *sag*, had been determined for multiple other *Mtvs*.

The limited information on *Mtv* sequence variation, phylogenetic relationships, genomic locations, and wild mouse distribution prompted us to re-examine their acquisition, diversity, and adaptive evolution in *Mus*. Here, we identified 29 *Mtvs* in the sequenced genomes of common mouse strains and wild mouse taxa, and we describe their integration sites, structural variations, and the shared ownership of specific *Mtvs*. The inbred strains carry different subsets of *Mtvs,* most of which were clearly acquired before the development of these strains. *Mtvs* have near-global distribution in *Mus musculus* subspecies but are not detectable in some European and Asian mice. Many *Mtvs* cloned from wild mice contain internal deletions, the most striking of which is a deletion in *env* that corresponds to an intron of the *rem* accessory factor gene, suggesting that this structure is derived from spliced MMTV cDNAs through recombination or gene conversion. We identified two recombination breakpoints in the highly variable *sag* gene along with evidence of positive selection, but the spread of potentially pathogenic MMTVs in wild mouse populations is not marked by genetic conflicts between the MMTV Env and the transferrin receptor it uses for entry (28).

## RESULTS AND DISCUSSION

We screened the genomic assemblies of 12 classical inbred strains, four *M. musculus* subspecies, and *M. spretus* (29) for MMTV-related sequences. We retrieved the three *Mtvs* in the C57BL/6 reference genome (*Mtv8, 9, *and *17*) (19) and identified 12 additional *Mtvs* in other laboratory strains and 14 in the wild mouse genomes (Table 1). These 29 *Mtvs* include 18 full-length genomes, four partially deleted *Mtvs*, one with two intermingled genomes, and six solo LTRs formed by homologous recombination between the flanking LTRs, which removes all intervening sequences leaving behind one LTR at the integration site. Twenty-one of the 22 2-LTR *Mtvs* contain coding sequence ORFs. Five of the six 2-LTR *M. spretus Mtvs* have ORFs for the protein-coding genes *gag*, *pro*, *pol*, *env,* and *sag*, whereas the *M. musculus Mtvs* are more defective, generally lacking an *env* ORF, although all but two have *sag* ORFs (Table 1). Mouse Genome Informatics (https://www.informatics.jax.org/) (30) lists 54 *Mtvs* defined in earlier studies by their diagnostic bands on Southern blots of which 19 were judged to be among our mined *Mtvs* based on strain distribution and chromosomal positions. New *Mtvs* are termed *Mtv55-64*. Sequences for all but the previously sequenced *Mtv1* are given in Datafile S1.

**TABLE 1:** *Mtvs* extracted from sequenced mouse genomes

Chr	*Mtv* ^*a*^	Strain^*c*^	Accession no.	Start	Size (bp)	Strand	TSD^*d*^	ORFs^*e*^					References^*f*^
								*gag*	*pro*	*pol*	*env*	*sag*	
Laboratory strain *Mtvs*													
1	7	DBA/2J	OW971804	175562065	9,979	+	TTCCT(A/C)	+	+	+	-	+	(31)
3	55	LP/J	OX390148	159170282	9,897	+	TCCCAT	+	+	+	-	+	This paper
4	17	C57BL/6J	NC_000070.6	46575144	9,901	+	GCTCCC	+	+	-	-	+	(32)
4	13	FVB/NJ	OW971712	104682372	9,851	-	AGGGAG	+	+	-	+	+	(33)
4	14^*b*^	CBA/J	OW971577	13777107	1,347	+	GAACTT	-	-	-	-	-	(21)
6	23	AKR/J	OW971599	15654865	9,901	+	TTAAAG	+	+	-	-	+	(21)
6	8	C57BL/6J	NC_000072.6	68242385	9,901	+	TTGTAC	+	+	+	-	+	(34)
7	1	C3H/HeJ	OW971859	42819164	9,851	-	GAAAAT	+	+	+	+	+	(8)
8	21	NZO/HILtJ	OX389802	14601162	9,898	+	CCTAGG	-	-	-	-	-	(32)
11	56	NZO/HILtJ	OX389805	1304749	8,468	+	ATATTC	+	+	-	-	+	This paper
11	3	NOD/ShiLtJ	OW971794	114032870	9,847	+	GTCTGC	+	+	-	-	+	(35)
12	9	C57BL/6J	NC_000078.7	92849314	9,901	-	AATAATC	+	+	+	-	+	(34)
14	11	C3H/HeJ	OW971849	53947657	9,901	+	GTTCTAACG	+	+	-	-	+	(36)
15	57	LP/J	OX390158	1123980	9,900	-	TCCATG	-	+	-	-	+	This paper
16	6	C3H/HeJ	OW971851	36628930	3,610	+	TCATCC	-	-	-	-	+	(34)
Wild mouse *Mtvs*													
1	58^*b*^	*M. spretus*	OW971678	105742622	1,300	+	GCTAAC	-	-	-	-	-	This paper
5	32	*M. spretus*	OW971684	78874630	9,932	+	CATTCC	+	+	+	+	+	(26)
6	33	*M. spretus*	OW971683	4145603	18,519	-	AAGAAG	+	+	+	+	+	(26)
6	34	*M. spretus*	OW971683	120370669	8,622	+		+	+	+	+	+	(26)
7	35	*M. spretus*	OW971685	128226642	6,888	-	ATGCTG	+	-	-	-	+	(26)
9	59^*b*^	*M. spretus*	OW971688	121213228	1,300	+	CCCCTG	-	-	-	-	-	This paper
15	36	*M. spretus*	OW971693	47727518	9,932	-	GAAGAA	+	+	+	+	+	(26)
16	37	*M. spretus*	OW971694	59583479	9,845	-	GATAAC	+	+	-	+	+	(26)
18	38	*M. spretus*	OW971696	66484462	9,911	+	TTTCCT	+	+	+	+	+	(26)
X	60^*b*^	*M. spretus*	OW971681	108760577	1,345	+	GATATC	-	-	-	-	-	This paper
X	61	CAST/EiJ	OW971844	61540140	9,932	+	ATCAAT	+	+	-	-	-	This paper
11	62	PWK/PhJ	OW971773	897278	9,899	-	GGAAGT	-	-	+	-	+	This paper
7	63^*b*^	WSB/EiJ	OW971628	99332558	1,329	+	CCCACT	-	-	-	-	-	This paper
13	64^*b*^	WSB/EiJ	0W971618	73254046	1,344	-	ACCATG	-	-	-	-	-	This paper

^*a*^ Genome locations and strain distribution identified 19 *Mtvs* as previously named *Mtvs* (*Mtv1-21,32–38*) (19, 22, 23) and 10 new *Mtvs* (*Mtv55-64*).

^*b*^ Solo LTRs

^*c*^ One strain is listed for each Mtv; chromosome assembly accession numbers and start positions are for that representative strain.

^*d*^ Target site duplication.

^*e*^ +, ORF present; −, ORF absent

^*f*^ References are for chromosome assignments for *Mtv* locus-specific restriction fragments.

Unlike other retroviruses, MMTVs show no integration site selection bias for particular genomic features (37). Some of the 29 *Mtvs* are inserted into sites marked by genomic repeats, deletions, or rearrangements that can complicate provirus identification by PCR (Table S1). Thus, *Mtv58* and *Mtv59* are both inserted into different positions in a long repeat identified by the Dfam repeat database (38) as GSAT_MM (also termed MaSAT, the major satellite), a 234 bp heterotetramer, which can be as long as 38 kb and represents 8% of the mouse genome (39). Other integration sites are flanked by polymorphic insertions or deletions including short stretches of simple G, GA, or CT repeats that can be truncated or absent from mice lacking the *Mtv* (*Mtv13, 38,* and *66*), translocated chromosomal segments found elsewhere in the B6 reference genome (*Mtv3, −7*), or a deletion spanning the integration site in *Mtv-11*-negative strains. In the four strains carrying *Mtv11*, the inserted 783 bp cellular sequence carries an unusually long 9 bp TSD (target site duplication) (Table 1).

### Distribution and wild mouse origins of inbred mouse *Mtvs*

**Fig 1:**
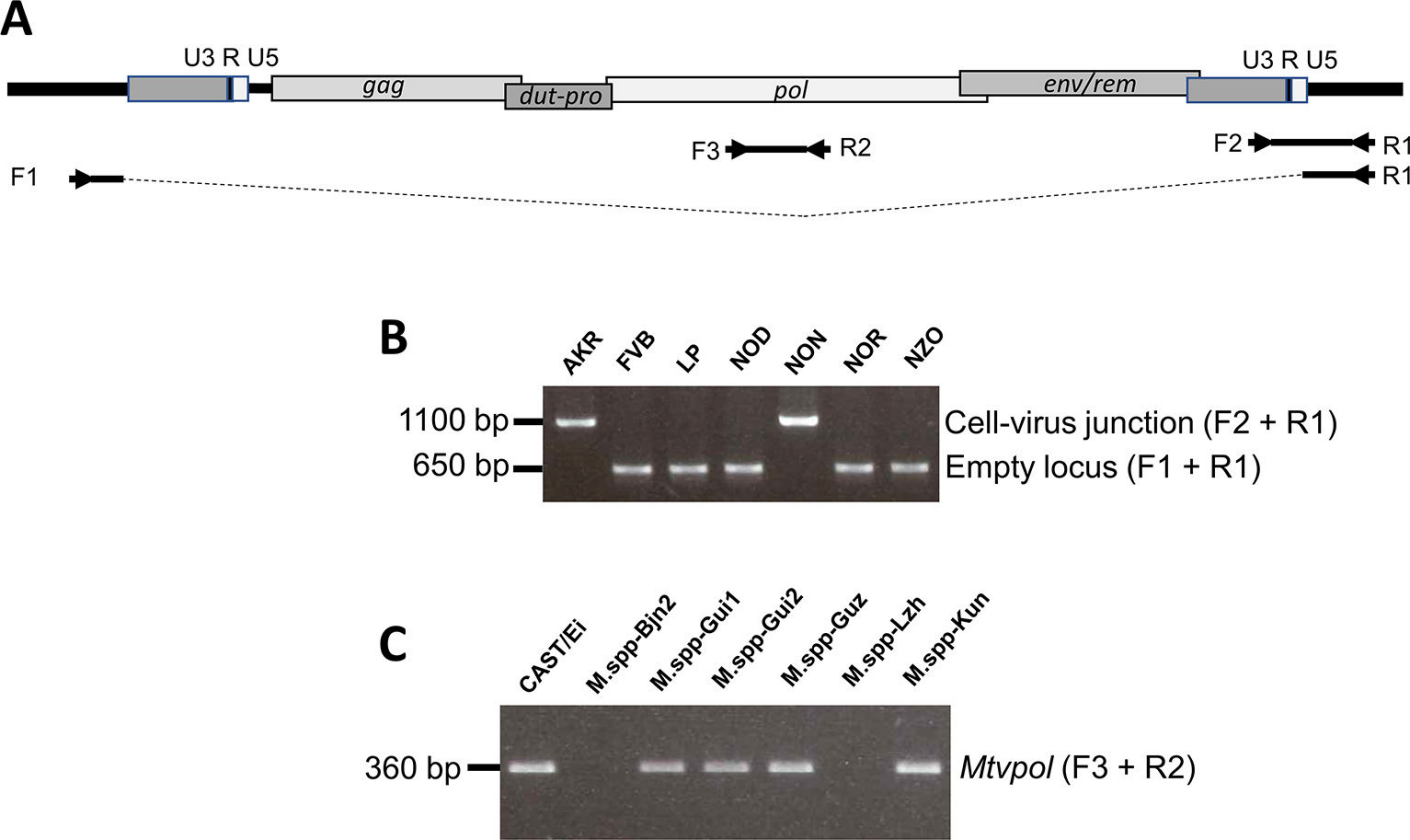
Identification of specific *Mtvs* and MMTV-related sequences in mouse genomes by PCR. (**A**) Arrows mark primer locations on the diagram of the MMTV provirus. Gel pictures illustrate expected size differences for cell-virus junction fragments versus the empty locus for *Mtv57* (**B**), and primers internal to the MMTV genome generate a *pol* product recruited from all *Mtvs* in that mouse (**C**).

Location-specific primers for 14 inbred strain *Mtvs* were used to amplify diagnostic cell-virus junction fragments or the empty locus to determine the distribution of each ERV in a panel of 37 inbred strains grouped according to their shared breeding history (Fig. 1; Table S2) (40). Individual *Mtvs* are shared by 3–27 strains typed by genomic screening or PCR. The classical inbred strain genomes are mosaics derived from fancy mice developed by hobbyists who interbred the three *Mus musculus* subspecies (*Mus musculus musculus*, *Mus musculus domesticus,* and *Mus musculus castaneus*) along with the natural hybrids of *musculus* and *castaneus* in Japan termed *M. m. molossinus* (41, 42). The classical inbred strains are therefore derived from a small gene pool, and the breeding histories of inbred strain lineages also involve significant cross-breeding. Although none of the *Mtvs* are fixed in all laboratory strains, some, like *Mtv8*, are broadly shared among the various lineages, whereas others have more restricted strain distributions: *Mtv1* is only found in Castle strains derived from founders obtained from breeder Abby Lathrop (43), and *Mtv21* is restricted to the New Zealand (NZ) strains (Table 2).

**TABLE 2:** Distribution of *Mtvs* in inbred mouse strains^*d*^

Strain groups^*a*^	Strains^*b*^	*Mtvs* ^*c*^														
		1	3	6	7	8	9	11	13^*d*^	14	17	21	23	55	56	57
C58/57	**C57BL/6J**	**-**	**-**	**-**	**-**	**+**	**+**	**-**	**-**	**-**	**+**	**-**	**-**	**-**	**-**	**-**
	C57BR/cdJ	**-**		**-**	**-**	**+**	**+**	**+**		**-**	**+**	**-**	**-**	**-**	**-**	**-**
	C58/J	**-**	**+**	**-**	**+**	**-**	**-**	**-**		**-**	**+**	**-**	**-**	**-**	**-**	**+**
	LT/SvEiJ	**-**			**+**	**+**	**-**	**-**		**-**	**+**	**-**	**-**	**-**	**-**	**+**
	C57L/J	**-**	**-**	**-**	**-**	**+**	**+**	**+**		**-**	**+**	**-**	**-**	**-**	**-**	**-**
	MA/MyJ	**-**		**-**	**-**	**+**		**-**		**-**	**+**		**-**	**-**	**-**	**-**
Castle	**1291/SvJ**	**-**	**-**	**-**	**-**	**+**	**+**	**+**	**+**	**-**	**+**	**-**	**-**	**+**	**-**	**-**
	**A/J**	**-**	**-**	**+**	**-**	**+**	**-**	**-**	**+**	**-**	**-**	**-**	**+**	**-**	**-**	**-**
	**AKR/J**	**-**	**-**	**-**	**+**	**+**	**+**	**-**	**-**	**-**	**+**	**-**	**+**	**-**	**-**	**-**
	**BALB/cJ**	**-**	**NB**	**+**	**-**	**+**	**+**	**-**	**-**	**-**	**-**	**-**	**-**	**-**	**-**	**-**
	**C3H/HeJ**	**+**	**NB**	**+**	**-**	**+**	**-**	**+**	**-**	**+**	**-**	**-**	**-**	**-**	**-**	**-**
	**CBA/J**	**-**	**NB**	**+**	**+**	**+**		**-**	**-**	**+**	**+**	**-**	**-**	**-**	**-**	**-**
	**DBA/2J**	**+**	**NB**	**+**	**+**	**+**	**-**	**+**	**+**	**+**	**+**	**-**	**-**	**-**	**-**	**-**
	F/St	**-**	**+**	**-**	**-**	**-**	**-**	**-**		**NB**	**+**	**-**	**-**		**-**	**-**
	I/LnJ	**-**	**-**	**-**	**+**	**-**	**-**	**-**		**-**	**+**	**-**	**-**	**-**	**-**	**+**
	**LP/J**	**-**	**NB**	**+**	**-**	**+**	**+**	**+**	**+**	**-**	**+**	**-**	**-**	**+**	**-**	**+**
	RF/J	**+**	**-**	**-**	**+**	**+**	**-**	**-**		**-**	**+**	**-**	**+**	**-**	**-**	**+**
	SEA/GnJ	**-**	**NB**	**+**	**+**	**+**	**-**	**-**		**-**	**-**	**-**	**-**	**-**	**-**	**-**
	SEC/1ReJ	**-**	**NB**	**+**	**-**	**+**	**+**	**-**		**-**	**-**	**-**	**-**	**-**	**-**	**-**
	SM/J	**-**	**-**	**+**	**+**	**+**	**-**	**-**		**+**	**+**	**-**	**-**	**-**	**+**	**-**
NZ	NZB/BINJ	**-**	**+**	**-**	**+**	**-**	**+**	**-**		**+**	**+**	**-**	**-**	**-**	**-**	**+**
	**NZO/HILtJ**	**-**	**+**		**+**	**-**	**-**	**-**	**-**	**+**	**+**	**+**	**-**	**-**	**+**	**-**
	NZM2410/J	**-**		**+**	**-**	**+**		**-**		**NB**		**+**	**-**	**-**	**+**	**-**
	NZW/LacJ	**-**	**+**		**-**	**+**	**-**	**-**		**NB**	**+**	**+**	**-**	**-**	**+**	**-**
Swiss	**FVB/NJ**	**-**	**-**	**-**	**-**	**+**	**-**	**-**	**+**	**+**	**+**	**-**	**-**	**-**	**-**	**-**
	NFS/N	**-**	**-**	**-**	**+**	**+**	**-**	**-**		**+**	**+**	**-**	**-**	**-**	**-**	**-**
	**NOD/ShiLtJ**	**-**	**+**	**-**	**-**	**-**	**-**	**-**	**-**	**-**	**+**	**-**	**-**	**-**	**-**	**-**
	NON/ShiLtJ	**-**	**+**	**+**	**+**	**-**		**-**		**+**	**+**	**-**	**+**	**-**	**-**	**-**
	NOR/LtJ	**-**	**+**	**-**	**-**	**-**	**+**	**-**		**NB**		**-**	**-**	**-**	**-**	**-**
	SJL/J	**-**	**-**	**-**	**-**	**+**	**-**	**-**		**-**	**-**	**-**	**-**	**-**	**-**	**-**
	SWR/J	**-**	**-**	**-**	**+**	**+**	**-**	**-**		**+**	**+**	**-**	**-**	**-**	**-**	**-**
Other	DDD	**-**	**NB**	**-**	**+**	**-**	**+**	**-**		**-**	**+**	**-**	**-**	**-**	**-**	**-**
	TALLYHO/JngJ	**-**	**NB**	**-**	**-**	**+**	**-**	**-**		**+**	**+**	**-**	**-**	**-**	**-**	**-**
	KK/HIJ	**-**	**+**	**+**	**-**	**-**	**-**	**-**		**-**		**-**	**-**	**+**	**-**	**-**
	LG/J	**-**	**-**	**-**	**-**	**+**	**+**	**-**		**+**	**+**	**-**	**-**	**-**	**-**	**-**
	PL/J	**-**	**+**	**-**	**-**	**+**	**-**	**-**		**-**	**+**	**-**	**+**	**-**	**-**	**-**
	RIIIS/J	**-**	**-**	**+**	**-**	**+**	**-**	**-**		**+**	**-**	**-**	**-**	**-**	**-**	**-**
																

^*a*^ Strain groups are based on (40).

^*b*^ Strains with sequenced genomes are bolded and underlined; all *Mtvs* in each of these strains are identified.

^*c*^ Highlighting indicates mice with *M.m.musculus* (light gray) or *castaneus* (dark gray) genomic substitutions spanning integration sites as determined by the Mouse Phylogeny Viewer ((http://msub.csbio.unc.edu); these produced the empty locus PCR product or failed to produce either the junction or empty locus amplicons. NB, no band.

^*d*^ Typing possible only for strains with sequenced genomes.

**Fig 2:**
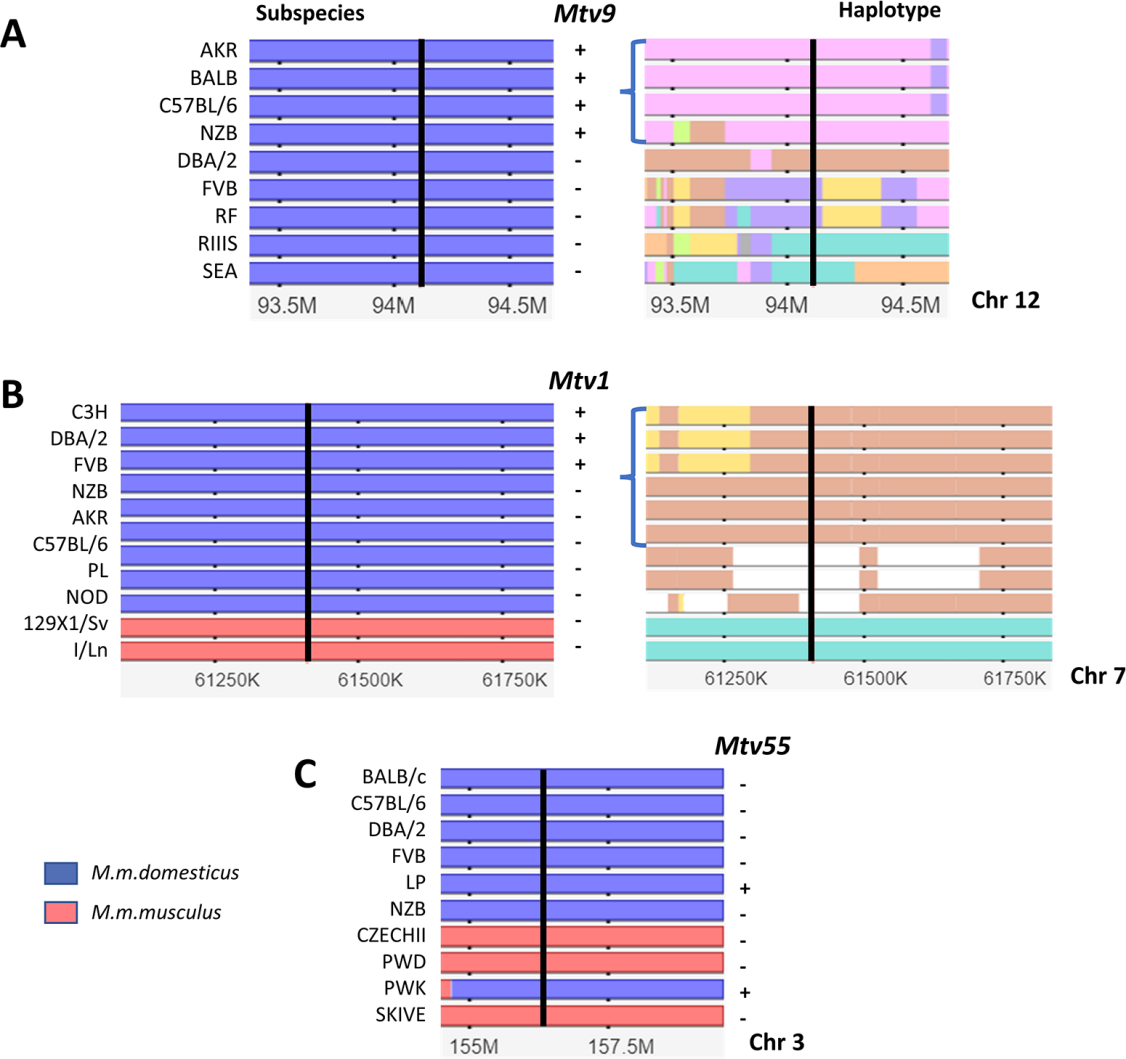
Subspecies origins and haplotype diversity at insertion sites for *Mtv9* (**A**) and *Mtv1* (**B**), and subspecies origins for *Mtv55* (**C**) as defined by the Mouse Phylogeny Viewer (44). The horizontal tracks represent chromosomal segments containing the insertion sites of the three *Mtvs,* which are marked by vertical black lines, using positions from NCBI37/mm9. All three panels contain images in which genomic regions originating from *M. m. domesticus* are in blue and from *M. m. musculus* in red, with PCR typings for each *Mtv* to the right of these images. Numbers below each image represent distance from the centromere in kb (panel B) or MB (panels A, C). In panels (**A**) and (**B**), the images to the right illustrate the different inbred strain haplotypes represented by arbitrary colors; brackets identify the haplotype containing the *Mtv. Mtv9* lies in a pink-colored haplotype shared by all four positive strains, whereas the *Mtv1* insertion site is only found in three of the six mice within the orange haplotype into which this *Mtv* was inserted. (**C**) The *Mtv55* insertion site lies in a *domesticus-*derived genome substitution in the PWK mouse.

Twelve of the 15 *Mtv* integration sites lie in regions covered by high-resolution genomic maps developed for the Mouse Phylogeny Viewer (MPV) database, which used single-nucleotide polymorphisms (SNPs) and variable intensity oligonucleotides (VINOs) to define the subspecies origins of segments along each chromosome (42). All 12 of these *Mtvs* are inserted into *M. m. domesticus*-derived segments of the genome as illustrated for three *Mtvs* in Fig. 2, suggesting that these *Mtvs* originated in *M. m. domesticus*.

Ninety-seven percent of the classical mouse genome derives from 10 haplotypes defined by linked SNPs and VINOs (42). Across inbred strains, specific haplotypes consistently contain the insertion sites of eight shared *Mtvs* (Table S3) as shown for *Mtv9* in Fig. 2A, suggesting their acquisition was prior to the development of inbred strains. The four exceptional cases (*Mtv1, 3, 13,* and *23*) are each embedded in specific haplotypes in some, but not all strains with that haplotype (Table S3), as illustrated for *Mtv1* (Fig. 2B). This suggests that these four *Mtvs* were acquired later than the other *Mtvs*. The first inbred strain, DBA, was developed in 1909, and the possibility that some *Mtvs* were acquired during early strain development accords with studies dating to 1907–1915 on the high incidence of mammary tumors in early laboratory colonies (45, 46).

### *Mtvs* in wild mouse taxa

The fact that most of the laboratory mouse *Mtvs* predate the origin of inbred strains suggests that they were acquired by fancy mice or their earlier wild mouse progenitors, and older studies identified MMTV-related sequences in *M. musculus* and *M. spretus* using Southern blotting (24–27). Here, we found five *Mtvs* in the sequenced genomes of three of four inbred wild-derived strains developed from *M. musculus* subspecies (Table 1): two in PWK/PhJ (*M. m. musculus*), one in CAST/EiJ (*M. m. castaneus),* two solo LTRs in WSB/EiJ (*M. m. domesticus*), and none in JF1/MsJ (*M. m. molossinus*). One of the two PWK *Mtvs* is identical to the inbred strain *Mtv55* by its genomic location and sequence identity. The second PWK *Mtv, Mtv62,* is embedded in a genomic substitution derived from *M. m. domesticus* (Fig. 2C), an example of the genomic contamination found in such wild-derived inbred strains through the inadvertent interbreeding of their wild-caught founders with laboratory mice after their introduction into the laboratory (42).

**Fig 3:**
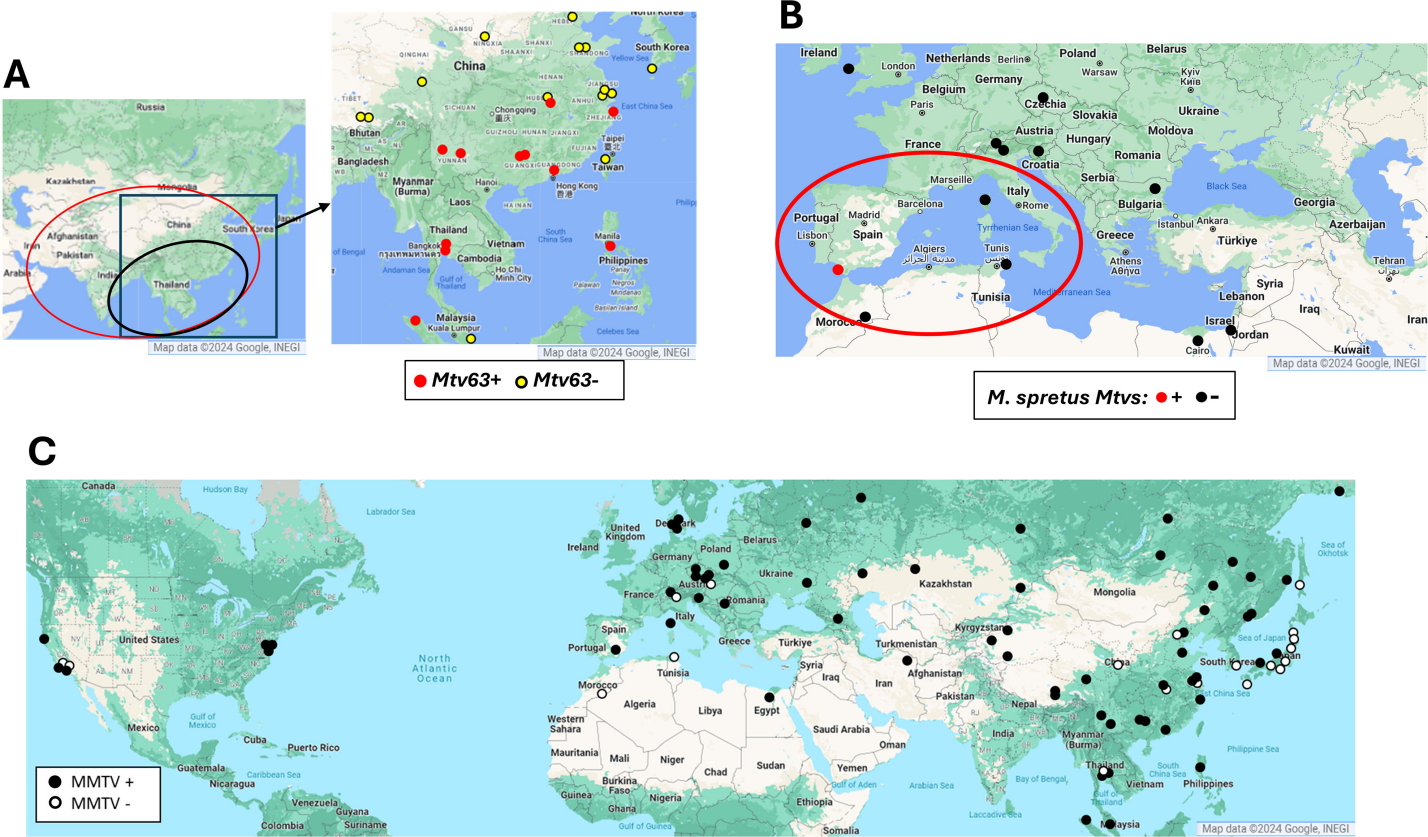
Geographic distribution of wild mice typed for *Mtv61* and the *M. spretus Mtvs*. (**A**) The red oval on the map to the left indicates the general range of *M. m. castaneus,* with a black oval locating one of its four sublineages (49, 50). The enlarged boxed area on the right shows the trapping locations of mice carrying *Mtv61*. (**B**) The range of *M. spretus* is marked by a red oval. Trapping locations are shown for mice typed for the *M. spretus Mtvs*. (**C**) Distribution of *Mtvs* in wild mice determined by typing for MMTV LTR, *gag*, *pol,* and/or *env* sequences. Negative mice were typed with at least four primer pairs and are concentrated in East Asia.

We screened a panel of DNAs from wild-caught mice (Table S4) for the *Mtvs* found in the laboratory and *M. musculus* strains to determine their geographic spread in wild mouse populations and taxonomic origins. The three *M. m. musculus* subspecies have generally well-defined, nonoverlapping ranges: *M. m. domesticus* in western Europe, *M. m. castaneus* in southeast Asia, *M. m. musculus* in eastern Europe to the Pacific, and *M. m. molossinus* in Japan (47). None of the 15 laboratory mouse *Mtvs* were identified in any wild-caught mice, but three wild mouse *Mtvs* were found in additional wild mice (Table 3). *Mtv63* and *Mtv64*, originally found in WSB/EiJ, were also detected in mice trapped at the same or a nearby location in Maryland (48). Most notably, *Mtv61* of CAST/EiJ was found in mice throughout SE Asia (Fig. 3A). The sequenced CAST/EiJ genome carries no other *Mtvs. M. m. castaneus* is a polytypic subspecies (49, 50) with a geographic range extending from SE Asia to the Iranian plateau, which is where *M. musculus* subspecies originated 0.25–0.5 MYA (51, 52). *M. m. castaneus* is more diverse than the *domesticus* or *musculus* subspecies, and its range contains at least four sublineages that, in the past, have been given a variety of alternative taxonomic designations and are here therefore labeled *M. musculus spp*. (Table S4) (49, 50). The geographic distribution of *Mtv61* corresponds closely to that of one of these sublineages (Fig. 3A).

**TABLE 3:** Distribution of individual *Mtvs* in inbred and wild mice

*Mtv*	Number positive/total tested				
	Inbred strains	*M. musculus* ranges^*a*^			
		Western Europe, Americas (*domesticus*)	Eastern Europe, North Asia (*musculus*)	South Asia (*castaneus*)	Japan (*molossinus*)
*1*	3/37	0/11	0/8	0/4	0/3
*3*	11/33	0/11	0/5	0/4	0/4
*6*	13/34	0/11	0/13	0/10	0/15
*7*	3/37	0/5	0/6	0/7	0/6
*8*	26/37	0/8	0/10	0/7	0/4
*9*	12/33	0/9	0/6	0/7	0/10
*11*	7/37	0/11	0/7	0/7	0/5
*14*	13/37	0/11	0/8	0/5	0/7
*17*	27/34	0/16	0/6	0/5	0/11
*21*	3/33	0/7	0/7	0/5	0/5
*23*	5/37	0/9	0/9	0/7	0/7
*55*	3/36	0/0	1/17^*b*^	0/4	0/4
*61*	0/24	0/13	0/11	10/33	0/13
*62*	0/35	0/9	1/20^*c*^	0/19	0/8
*63*	0/21	2/24	0/12	0/4	0/4
*64*	0/35	1/25	0/2	0/7	0/1

^*a*^ Subspecies assignments based on trapping sites. *Mtvs13*, -*56,* and -*57* could not be reliably typed in wild mice. *Mtvs* found in additional wild mice are underlined.

^*b*^ PWK *Mtv* is shared by PWD, a wild-derived strain also trapped in the former Czechoslovakia.

^*c*^ Identified within a genomic contaminant of PWK from *M. m. domesticus* (Fig. 2C).

The only taxon carrying *Mtvs* outside the house mice complex, *M. spretus,* diverged from *M. musculus* 2 MYA (53). *M. spretus* is sympatric with *M. m. domesticus* in northern Africa, Spain, and France. These taxa are interfertile, and although they occupy different ecological niches, bidirectional introgression between them has been documented in the wild, with gene flow predominantly from *domesticus* to *spretus* (54, 55) . None of the 10 SPRET/EiJ *Mtvs* were found in samples of *M. m. domesticus* from sympatric or bordering areas in N. Africa, Spain, and France (Fig. 3B). Our failure to find evidence of introgression may be due to limited sampling, but it is also possible that the *spretus Mtvs* could have been acquired from *M. musculus* through infection, as an MMTV XRV has been isolated from at least one European mouse (CZECHII/EiJ) (56).

Finally, to get a fuller picture of the diversity and distribution of *Mtv*s in wild mouse populations, we screened our wild mouse panel by PCR for MMTV LTR, *gag*, *pol*, and *env* segments. *Mus* taxa other than *M. musculus* and *M. spretus* do not carry such sequences including *M. spicilegus*, *M. fragilicauda,* and *M. macedonicus*, sister species of *M. spretus*. Such MMTV sequences, however, were found throughout the global range of *M. musculus*; the few exceptions include some individual mice of *M. m. musculus*, *M. m. castaneus,* and most Japanese mice (Fig. 3C). This is consistent with previous DNA hybridization tests of a more limited set of mice (24, 26) and is not surprising for populations that carry a low number of *Mtv* copies that are insertionally polymorphic.

**Fig 4:**
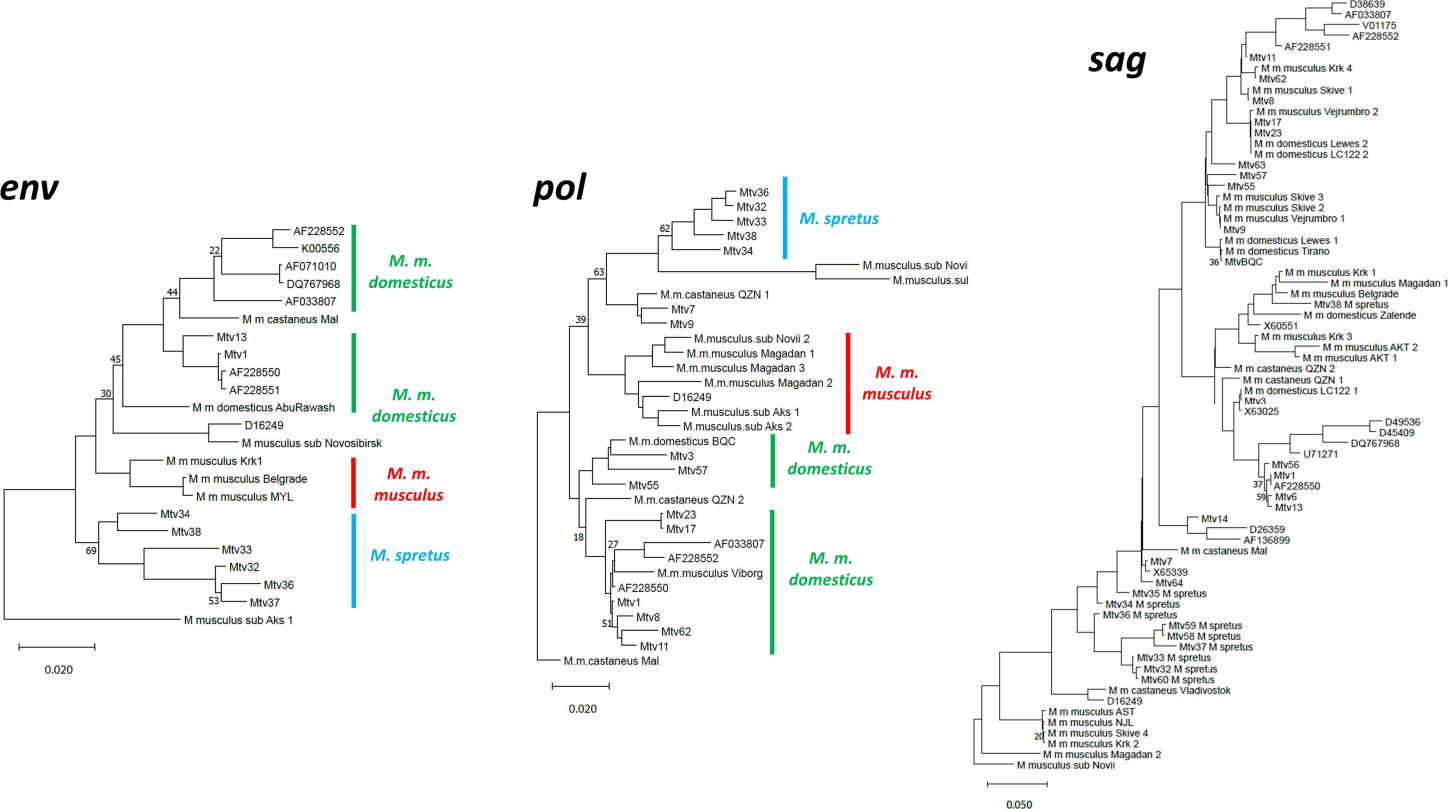
Phylogenetic analysis of *Mtv env, pol,* and *sag* genes. A maximum likelihood tree was generated with 500 bootstraps. The trees were midrooted. The scale bar represents 0.02 (for *env* and *pol*) or 0.05 (for *sag*) nucleotide substitutions per site. Tips with accession numbers represent previously sequenced *Mtvs* (Table S5). Vertical lines identify clusters based on source taxa or trapping location: green, *M. m. domesticus*; red, *M. m. musculus*; blue, *M. spretus*.

The *pol*, *env,* and *sag* sequences cloned from wild mice or extracted from genome assemblies were aligned with previously sequenced MMTV genes (Table S5) and used to construct phylogenetic trees (Fig. 4). The *pol* and *env* sequences, but not *sag*, generally fall into clades that confirm the relatedness of the majority of laboratory mouse MMTV ERVs and XRVs with those of *M. m. domesticus*, with separate groupings for Eurasian MMTVs and the *M. spretus Mtvs*.

### Structural *Mtv* variants include recurrent deletion of a *rem* intron

**Fig 5:**
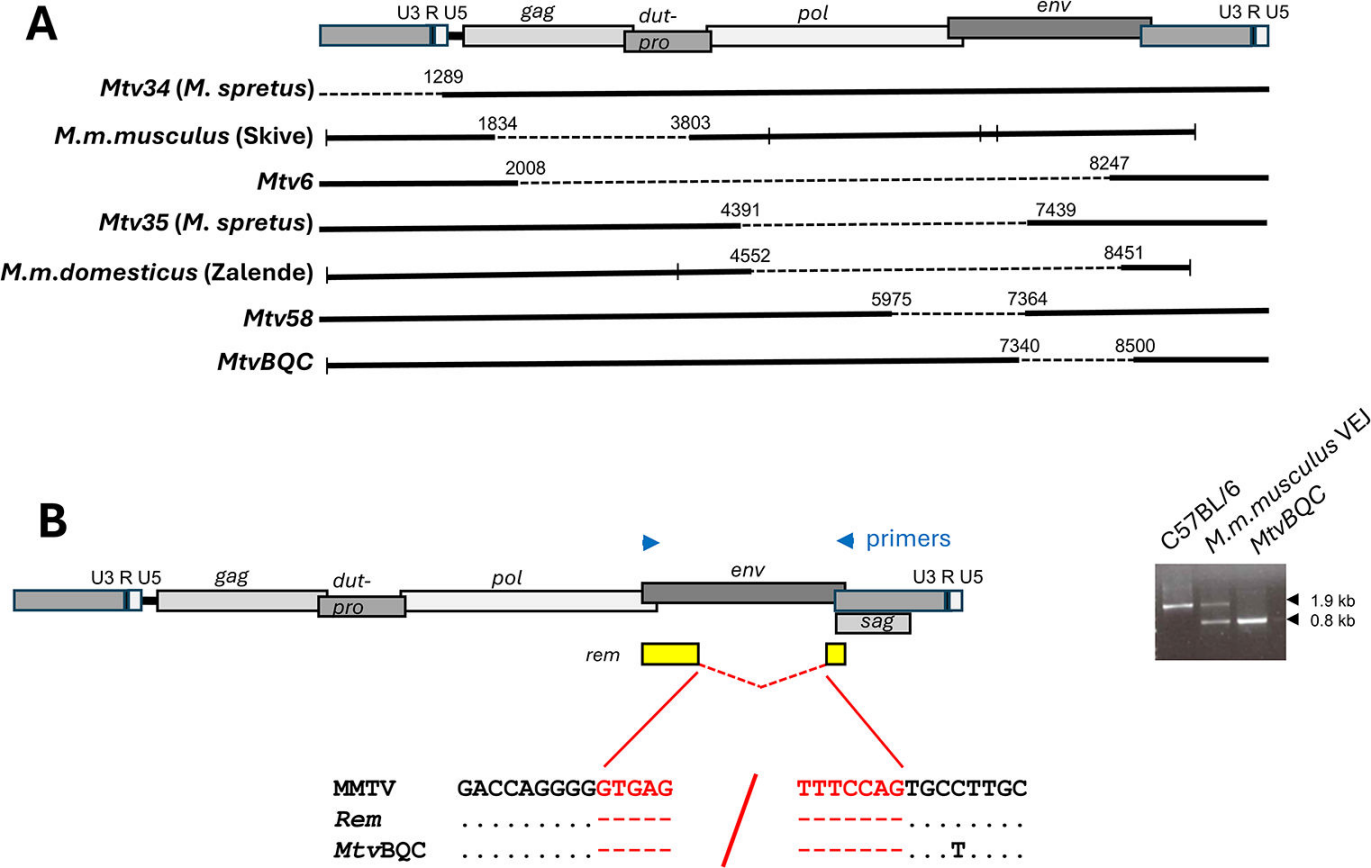
Large deletions include an *env* deletion corresponding to the *rem* intron. (A) Seven deletions were identified in *Mtvs* from sequenced genomes or from proviruses cloned from wild mice. Positions for each deletion are based on the *Mtv1* genome (GenBank No. AF22850). Short vertical lines in the three newly sequenced *Mtvs* represent locations for the PCR primers used to amplify segments for sequencing. (B) Blue arrows above the *Mtv* genome position the PCR primers that generate bands as shown in the gel picture on the right. *M. m. musculus* VEJ is a wild-derived mouse from Vejrumbro, Denmark that carries *Mtvs* with normal and deleted *env* genes. Yellow boxes show coding sequences for *rem* below, which are sequences flanking the splice sites in undeleted MMTV *env* genes, the *rem* cDNA (GenBank number DQ223969), and the provirus *MtvBQC*, an otherwise full-length *Mtv* with a provirus-like LTR (GenBank number PQ434773).

The 29 *Mtvs* and PCR-amplified MMTV sequences include six with internal deletions and one with a 5’ truncation (*Mtv34*) (Fig. 5). Six of the seven *Mtvs* with internal deletions remove different segments of the viral genome of 1160–6239 bp.

The seventh and most interesting deletion removes a segment of *env* and was found in 14 wild mice, but not in any inbred strains. This in-frame deletion exactly corresponds to an intron removed in the generation of the Rem accessory factor (16, 17). Such an ERV *env* structure most likely derives from cDNAs of spliced MMTV mRNAs, which then engage in either recombination with copackaged MMTV genomes, gene conversion, or LINE1-mediated retrotransposition. This deleted ERV is the only *env* structure in a wild mouse trapped in Bouquet Canyon, CA (BQC), and the sequence of this provirus and its cellular flank show that the deleted *env* is embedded in an otherwise full-length BQC *Mtv* with provirus-like LTRs and therefore does not represent a retrotransposed partially spliced *rem* cDNA. The cellular sequence flanking the BQC provirus is the repetitive element GSAT-MM (Datafile S2), making it the third *Mtv* inserted into this repetitive sequence. This major satellite has a pericentromeric location on the different mouse chromosomes; hence, the *MtvBQC* chromosomal location could not be identified.

**Fig 6:**
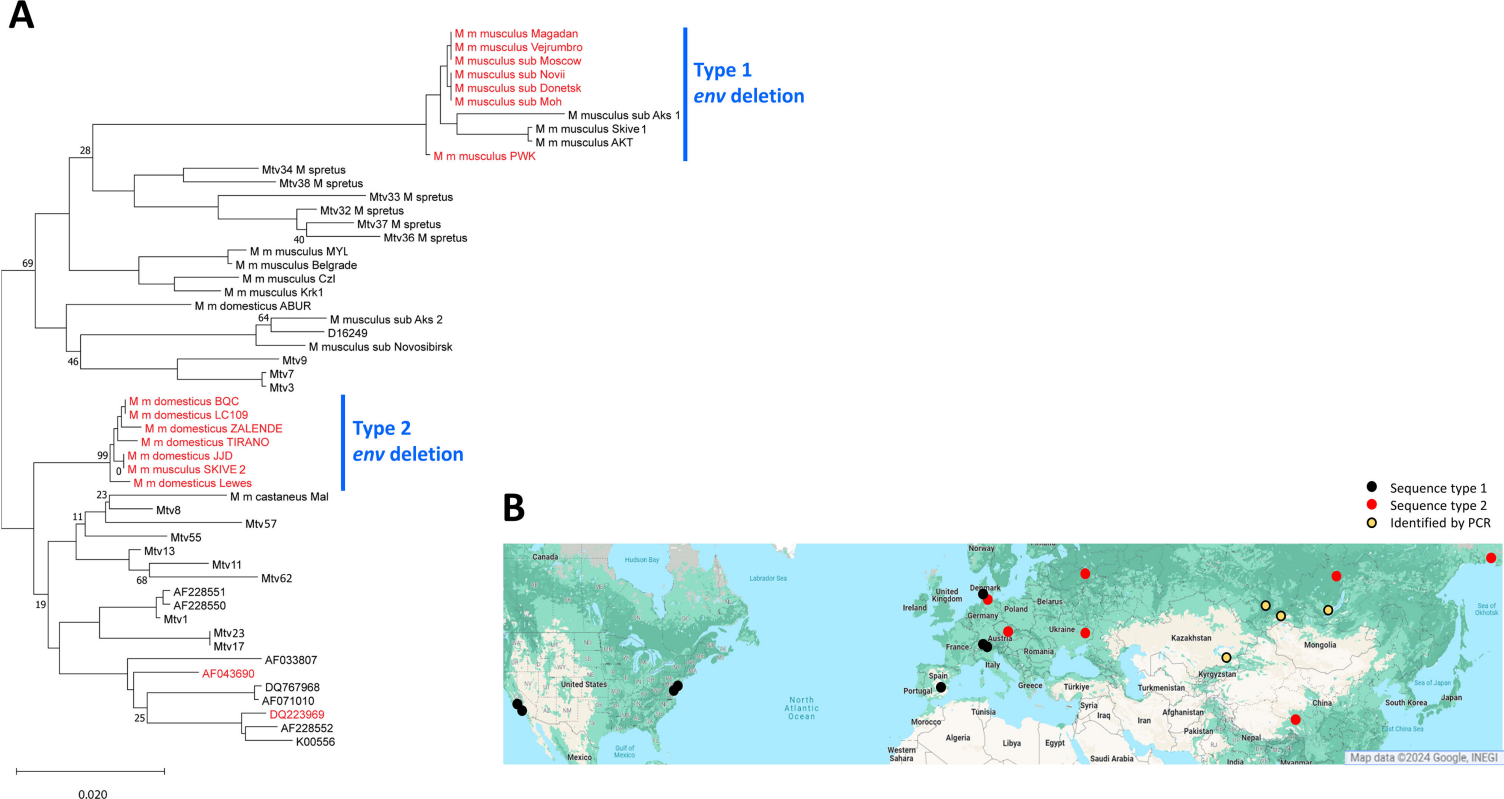
Phylogenetic tree and geographic distribution of *Mtvs* with the *rem* intron deletion. (**A**) *Mtv env* genes were aligned, and a maximum likelihood tree was generated with 500 bootstraps. The tree was midrooted. The scale bar represents 0.02 nucleotide substitutions per site. Deleted *env* genes are in red as are two GenBank sequences for *rem* cDNAs. Other accession numbers represent previously sequenced *Mtvs* (Table S5). Vertical lines identify two distinct sequence types of deleted *env* genes. (**B**) Geographic distribution of mice carrying the *env* genes with the *rem* intron deletion. The two *env* sequence types are represented by red and black circles. Yellow circles identify mice with deleted *envs* that were not sequenced.

There are two sequence variants among the 14 *env* genes with this deletion, designated Type 1 and Type 2. On a phylogenetic tree of *env* genes, these variants define separate clades that group, respectively, with *musculus* or *domesticus env* sequences (Fig. 6A). The trapping sites of the wild mice carrying the two types of this deleted *env* are broadly distributed and correspond to the ranges of *domesticus* (Type 1) and *musculus/castaneus* (Type 2) (Fig. 6B). The one exception to the clade/subspecies correspondence, *M. m. musculus*-SKIVE, with a *domesticus-*like *Rem,* was trapped near the hybrid zone separating the ranges of these mice in Europe (57). Gene introgression across this zone is mainly west to east (58), suggesting the acquisition of this *Mtv* through interbreeding with *M. m. domesticus*. The widespread distribution and subspecies-specific sequence variation of this deleted *env* coupled with the fact that the *rem* deletion is not shared by all mice in the same lineage indicates that its generation is a recurrent process rather than the amplification of a rare ancestral event.

Examples of spliced retroviral insertions are rare, but possible in principle since the cDNA of a Ty retroelement construct containing a spliceable intron was able to mediate gene conversion of a homologous copy in yeast (59, 60). A naturally occurring example of this process was reported in the human genome among HERV-H elements (61). The most common form of HERV-H carries four large deletions that produce an unusual spliced RNA using a cryptic splice site; this spliced form was identified among a set of cloned HERV-H proviruses. The present study identifies the first ERVs containing the spliced form of a functionally important viral coding gene, *rem*.

The frequent generation of ERVs containing intron-deleted *rem* raises the possibility that these ERVs may be selected for a useful host function while preventing *env* expression, which can be detrimental. All *env* genes with the deletion retain ORFs for Rem while losing the ability to produce Env. In contrast, most *env* genes in full-length *Mtvs* are defective (Table 1). In fact, many human ERV groups are marked by widespread *env* deletions, and such deletions likely protect the host from the negative consequences of retroviral *env* expression such as detrimental syncytia formation, downregulation of the cellular protein used as a receptor, and production of infectious virus. As for the possible benefit of a retained *rem* ORF, we cannot assess the function of these deleted *env* genes in the long-dead mice used here for DNA extraction. However, the 98 amino acid signal peptide of Rem shares the same function as the accessory proteins Rev and Rec, respectively, produced by HIV-1 and HERVK (HML-2), which are also spliced from *env*. All three of these proteins are responsible for the nuclear export of unspliced viral mRNAs. Although it is unlikely that this capability would benefit host cells, it is intriguing that the functionally related Rec also activates an innate antiviral response in embryonic stem cells by increasing levels of the viral restriction factor IFITM1, potentially inhibiting various IFITM1-sensitive viruses during embryogenesis (62).

### Phylogenetic analysis of the MMTV *env* and the gene encoding its cellular receptor

MMTVs use the transferrin receptor (Tfrc) to enter cells (28). This protein is also used as a receptor in multiple species by New World rodent arenaviruses and by some parvoviruses and malarial parasites (63–65). Arenaviruses bind to a different site on the receptor than do MMTVs (66, 67) but are not known to infect mice.

A previous study found that the *Tfrc* gene of seven rodent species is under positive selection that targets the receptor sites used by MMTV and arenaviruses (68). Although some rodent species other than house mice carry defective, distantly related MMTV ERVs (68), active infectious and pathogenic MMTVs are confined to mice; hence, we looked for possible co-evolutionary patterns in the interactive segments of MMTV Env and Tfrc in *Mus* taxa, emphasizing *Mtv*-positive mice. Such co-evolution can be identified by positive or diversifying selection determined by a high ratio of nonsynonymous mutations relative to synonymous mutations.

Analysis of the full-length *Tfrc* genes from 12 wild or inbred strains of *M. musculus* and 10 other species belonging to the *Mus* genus identified six sites under positive/diversifying selection (Table 4), but five of these sites are in or just outside of the arenavirus binding sites suggestive of genetic conflicts with these viruses. The sixth site was upstream of one of the two segments of *Tfrc* critical for MMTV binding (67), but this site, 564A, has not been implicated in entry (Fig S1). Cloned *Trfc* segments carrying the MMTV binding sites from nine additional wild *M. musculus* mice included no additional sequence variants (Datafile S3). Analysis of the MMTV *env* found no sites under positive selection, and no sequence variants around the previously defined receptor binding site (69), although structural studies suggest other, unidentified Env sites may function to affect MMTV entry (70). These data indicate that the acquisition, spread, and adaptation of MMTV ERVs in *Mus* taxa has not been accompanied by the generation of viral host range variants and altered receptors.

**TABLE 4:** Positive selection of sites in the Tfrc receptor and the MMTV Env

Gene	Codon frequency	ω^0^	M7-M8		Treelength	dN/dS (%)	Residues with dN/dS > 1, pr >0.95
			2δ	*P* value			
MMTV Env	f3 × 4	0.3	5.35	0.07	1.30758	N.S.^*b*^	N.S.
	f3 × 4	1.7	5.35	0.07	1.30758	N.S.	N.S.
Transferrin receptor	f3 × 4	0.3	25.47	0.000003	0.33277	9.01 (1.1)	199G^*a*^ 207Q*, 211N, 212L*, 354S, 564A
	f3 × 4	1.7	25.47	0.000003	0.33277	9.01 (1.1)	199G^*a*^ 207Q*, 211N, 212L*, 354S, 564A

^*a*^ pr >0.99%.

^*b*^ N.S., not significant.

### Recombination and positive selection of *sag*

Sag proteins were implicated as having antiviral activity when map locations of *Mtvs* were found to coincide with the *Mls* genes (minor lymphocyte stimulating antigens) (71). The C-terminus of Sag is highly variable and interacts with the variable region of the T cell receptor β-chain to eliminate cells expressing that Vβ gene, protecting mice from infection by MMTVs with the same Sag specificities (15). Thus, this LTR-encoded ERV protein is a member of the set of virus-derived antiviral proteins that, in mice, include Fv1, Fv4, and Rmcf1,−2 (72), and the importance of *Mtv*-encoded Sag to the host is underscored by the fact that only two of the 2-LTR *Mtvs* lack a *sag* ORF (Table 1).

**Fig 7:**
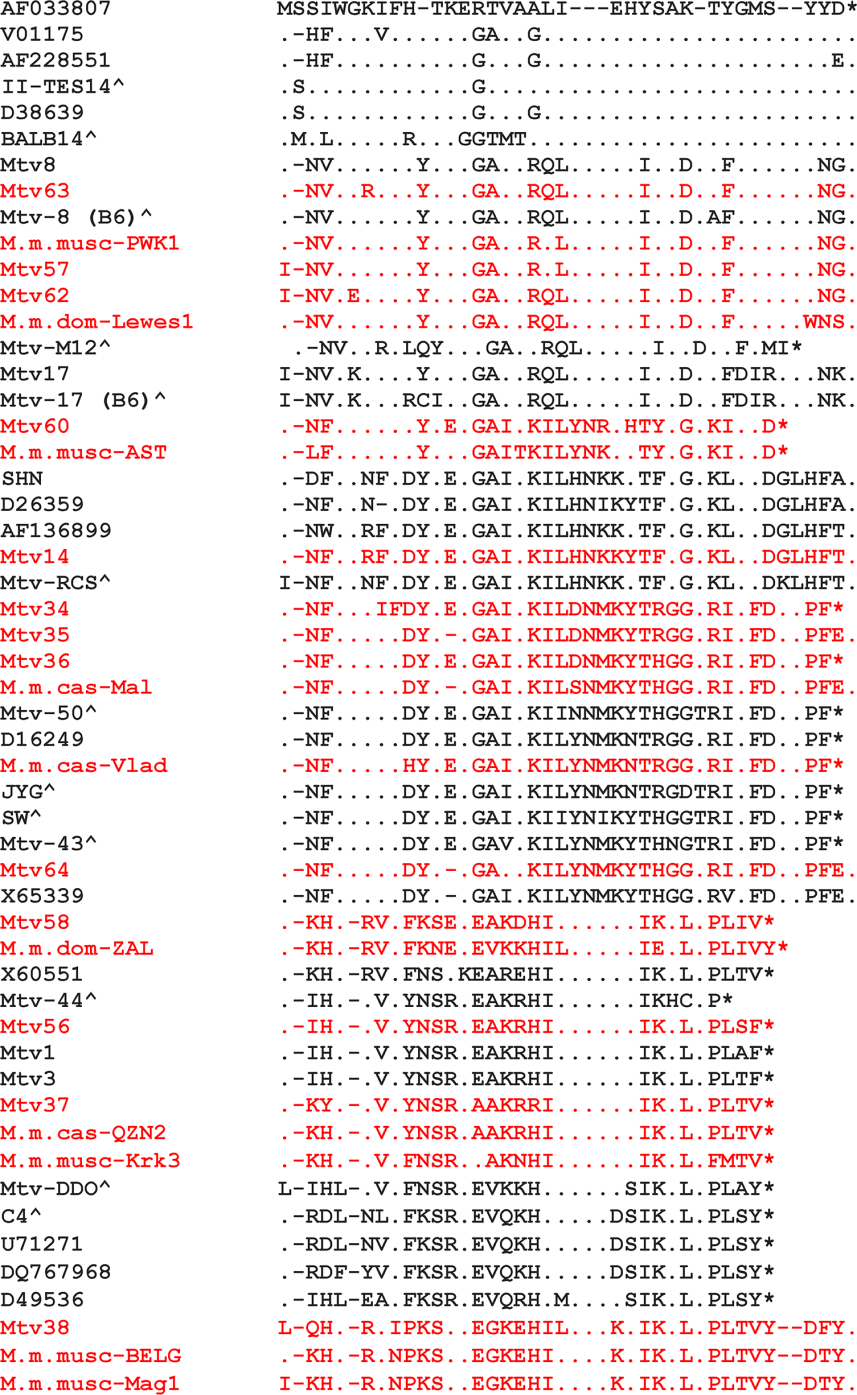
Sequence variants of the C-terminus of the MMTV *sag* gene. Newly identified variants are in red and were obtained from mined *Mtvs* or PCR products from wild mice (GenBank No. 434792–4347820). Other sequences were obtained from a previous compilation (marked with a ^) (73) or are identified by GenBank accession number. -, deletion; ., identical residue; *, stop codon.

Analysis of *sag* genes extracted from sequenced mouse genomes or cloned from wild mice identified 24 additional variants of the Sag C-terminus (Fig. 7). As pointed out, these variants do not cluster in clades that reflect the relationships of host mice (Fig. 4). In fact, 16 of the 17 sequenced mouse genomes carry *Mtvs* with more than one *sag/Mls* specificity. Mice that have acquired multiple *Mtvs* with different *sag* genes can inhibit multiple MMTV XRVs and therefore should have a clear fitness advantage.

**Fig 8:**
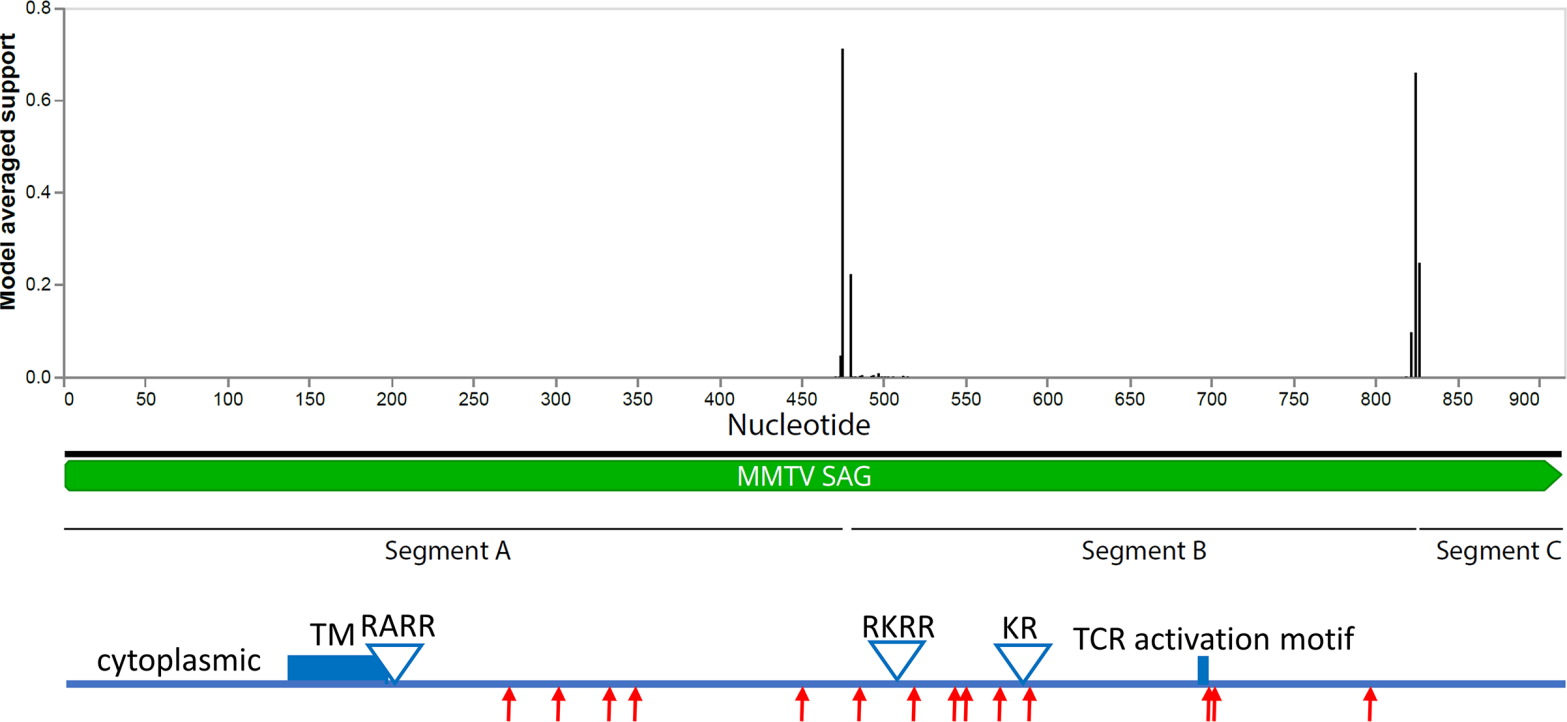
Analysis of MMTV *sag* genes for evidence of recombination. (Top) Plot with the model-averaged support (y-axis) for recombination breakpoints as calculated by GARD is shown above the *sag* gene. Nucleotide positions are indicated on the x-axis. (Bottom) Diagram of the *sag* gene identifying key motifs including the transmembrane domain (TM), three proteolytic cleavage motifs, and the TCR activation site (76, 77). Red arrows identify sites under positive selection.

Because host restriction factors commonly engage in an arms race with the pathogens they target, we evaluated *sag* genes for evidence of positive selection. A previous study found that separating the C-terminus from the rest of the *sag* sequence produced two significantly different phylogenetic trees (74); hence, we first examined *sag* for evidence of recombination because recombination can produce false signatures of selection (75). This analysis identified two recombination breakpoints dividing the gene into three segments (A–C) (Fig. 8). The trees produced from each segment differ from each other and, like the full-length tree (Fig. 4), do not show clear taxon correlations (Fig S2 ). The A and B segments each had multiple sites under positive selection including 2 and 3 sites, respectively, at 99% significance, whereas segment C, encoding the variable C-terminus, showed no codons under positive selection (Table 5). Although the sites under positive selection have not been assigned specific functional roles, two abut the TCR activation motif (76) (Fig. 8).

**TABLE 5:** Positive selection of sites in three Sag segments

Gene	Codon frequency	ω^0^	M7-M8		Treelength	dN/dS (%)	Residues with dN/dS > 1, pr >0.95
			2δ	*P* value			
MMTV Sag Segment A	f3 × 4	0.3	25.36	0.000003	2.15072	2.65(11.5)	90D, 101G, 113P^*a*^ 116S, 150P*
	f3 × 4	1.7	25.36	0.000003	2.15072	2.65(11.5)	90D, 101G, 113P^*a*^ 116S, 150P*
MMTV Sag Segment B	f3 × 4	0.3	26.61	0.000002	3.43469	1.68 (10.9)	160R^*a*^ 173T, 181G, 183R*, 191R, 196A, 233F, 234D, 264M*
	f3 × 4	1.7	26.61	0.000002	3.43469	1.68 (10.9)	160R^*a*^ 173T, 181G, 183R*, 191R, 196A, 233F, 234D, 264M*
MMTV Sag Segment C	f3 × 4	0.3	0.202	0.904125	14.4531	N.S.^*b*^	N.S.
	f3 × 4	1.7	0.202	0.904125	14.4531	N.S.	N.S.

^*a*^ pr >0.99%

^*b*^ N.S., not significant.

### Conclusions

Here, we extracted and analyzed 29 *Mtvs* found in the 17 sequenced mouse genomes along with additional variants cloned from wild mice, describing their sequence variation and their strain and wild mouse distribution. Most notably, we found a high degree of *Mtv* insertional polymorphism in inbred and wild mice, documented the recurrent generation of *Mtvs* with an *env* deletion corresponding to an intron in the *rem* accessory factor gene, showed *sag* genes to be modified by recombination and positive selection, but found no evidence of an arms race between the *Mtv env* gene and that of its entry receptor.

These data show that the acquisition of MMTV ERVs is a recent event in *Mus* evolution based on their insertional polymorphism and absence in taxa other than *M. musculus* and *M. spretus*. Their presence in all *M. musculus* subspecies indicates that this virus was circulating at the time and place of subspeciation. Although none of the 15 laboratory mouse *Mtvs* could be traced to specific wild mouse progenitors, examination of the subspecific origins of the insertion sites of the *Mtvs* of laboratory mice indicates that they all originated in *M. m. domesticus*, which is generally confirmed by the phylogenetic relationships defined by their *env* and *pol* genes.

The observed pattern and timeline of *Mtv* acquisition are roughly comparable with that of the MLVs, pathogenic gammaretroviruses, which are also only found in *M. musculus* and *M. spretus* (78). This nearly identical distribution of MLV ERVs and *Mtvs* in wild mouse taxa suggests they were both acquired at or just prior to *M. musculus* subspeciation. Endemic pathogens and their hosts are often subjected to bidirectional selective pressures resulting in cyclical patterns of evasion and counter-adaptation by virus and host, but the adaptive paths taken by these two retroviruses in the same hosts during the same time frame show significant differences. In particular, the Eurasian *Mus musculus* subspecies carry six functional variants of the MLV XPR1 receptor that differentially support entry of five Env-determined host range MLV subgroups (79). In contrast, the interacting sites of Tfrc and the MMTV Env have remained unchanged during this same relatively short evolutionary timespan, a stasis also seen in the unaltered *env* of ecotropic MLV XRVs that use the similarly unaltered CAT1 receptor, instead relying on the *Fv4* restriction gene to block virus entry (80). The failure to develop adaptive changes at the interface of the MMTV Env and its receptor suggests that other host factors, like co-opted *sag* genes or integrated copies of processed *rem* genes, may operate efficiently to mitigate the consequences of potentially lethal virus challenge.

## MATERIALS AND METHODS

### Sources of mouse DNAs and RNAs

Sources of mice and DNAs are listed in Table S4. Some DNAs were isolated from mice maintained in our laboratory or from mice obtained from S. Rasheed (University of Southern California, Los Angeles) and M. Potter (NCI, Bethesda, MD). Additional mice or DNAs from wild-caught or wild-derived mice were purchased from The Jackson Laboratory (Bar Harbor, ME) and from RIKEN BioResource Center (Tsukuba, Japan) with the assistance of T. Shiroishi and T. Murata. Mouse DNAs were also provided by R. Abe (Naval Medical Research Institute, Bethesda, MD), R. Elliott (Roswell Park Cancer Institute, Buffalo, NY), and S. Chattopadhyay and H. Morse III (NIAID, Bethesda, MD). Cell lines developed from some wild mice were obtained from J. Hartley (NIAID, Bethesda, MD).

### Genome screening for *Mtvs*

*Mtv* proviral copies were extracted from the latest assemblies of 12 classical inbred mouse strains, four wild-derived *M. musculus* strains, and *M. spretus* (29) obtained through NCBI (81). The genomes were screened using as probes *Mtv1* (GenBank No. AF228550) and two MMTV XRVs (GenBank Nos. AF228550 and AF033807) using BLAST (blast.ncbi.nlm.nih.gov) (82) and the BLAST/BLAT tools at Ensembl, release 112 (83).

### Cloning and sequencing *Mtvs*, *Mtv* flanks, and *Tfrc*

Primers for PCR (Table S2) were designed to amplify segments of the MMTV genome from genomic mouse DNAs, cell-virus junction fragments, empty insertion sites, the full-length *Tfrc* receptor gene, or genomic *Tfrc* segments that include exons 8 and 17, which encode the receptor determining regions. Asian mice that failed to produce MMTV PCR products using primers based on laboratory mouse *Mtvs* were also tested with primers specific to the CAST/Ei *Mtv61*.

Selected PCR products were cloned into pCR2.1-TOPO (Thermo Fisher, Waltham, MA) and sequenced in-house or by Psomagen (Rockville, MD). Other sequences used for analysis included previously reported mouse ERVs and XRVs (Table S5).

The *MtvBQC* cell-virus junction was cloned using inverse PCR (84): BQC mouse DNA was digested with TaqI (virus position 8985), circularized with ligase, and linearized with PstI (virus position 9503). The three enzymes were purchased from New England Biolabs (Ipswich, MA). The linearized fragment was amplified with primers flanking the PstI site in opposite orientations to each other (5′-GCCTTTATGAGCCCAACCTTGC, 5′-GGCGAGTCTTTCACGGAAGG), then cloned and sequenced.

### Identification of the subspecies origin and haplotype identity of laboratory mouse *Mtvs*

We used the Mouse Phylogeny Viewer (MPV) at the University of North Carolina (http://msub.csbio.unc.edu) (85) to identify the subspecies of origin and shared haplotypes of *Mtvs*. Searches were done using positions of the various *Mtv* flanking sequences in the NCBI37/mm9 reference assembly as identified by BLAT searches (86) using the UCSC genome browser (http://genome.ucsc.edu/).

### Phylogenetic trees, recombination, and positive selection analyses

The sequence of the *Tfrc* gene and segments from MMTV ERVs and XRVs including full-length and *rem* intron-deleted *env* genes, the reverse transcriptase domain of *pol*, and the *sag* gene were aligned using MUSCLE as implemented in Geneious 10.0.9 using default settings (87, 88). The analysis used mined *Mtvs* (Table 1) except for the degraded *Mtv61* and *Mtv21*, newly sequenced wild mouse ERVs, and previously published MMTV ERVs and XRVs (Table S5). Phylogenetic trees were generated using the RaxML program with the GTR + G + I model and 500 bootstraps for branch support (89).

For maximum-likelihood analysis of codon evolution, we used codeml of PAML 4.9 (90). Alignments for the intact *env* and *sag* genes of MMTVs and *Tfrc* genomic segments were trimmed to the shortest sequence and were manually inspected to exclude indels, as recommended by the developers of PAML. Primer sequences used for amplification were excluded from the analysis. To identify specific codons under positive selection, the F3 × 4 codon frequency model was used with different initial seed values of ω. Likelihood ratio tests were performed to compare M7, a neutral model with beta distribution of *dN*/*dS* values, with M8, a positive-selection model with beta distribution. In each case, χ^2^ analysis was done, and significance was determined with *P* < 0.01. Bayes Empirical Bayes algorithm in the M8 model was used to infer posterior probabilities of codons under positive selection (91).

To test for evidence of recombination in the *sag* gene, we utilized the GARD program (92) from the datamonkey webserver (93) using the general discrete model of site-to-site rate variation with two rate classes. Segments generated by GARD were independently assessed for positive selection.

## Data Availability

Sequence data were deposited to GenBank for full-length *Mtvs* under accession numbers PQ434773 to PQ434775, for the transferrin receptor under PQ434776 to PQ434791, for the *Mtv sag* gene under PQ434792 to PQ434820, for *Mtv pol* under PQ434821 to PQ434832, and for *Mtv env* under PQ434833 to PQ434848 and PQ434850 to PQ434857. All other data are available without restriction upon written request to the corresponding author.

## SUPPLEMENTAL MATERIAL

Data File S1 jvi.00079-25-s0001.txt Sequences for *Mtvs* extracted from 16 sequenced mouse genomes.

Supplemental material jvi.00079-25-s0002.pdf Figures S1 and S2, Tables S1 to S5, and Data Files S2 and S3.

ASM does not own the copyrights to Supplemental Material that may be linked to, or accessed through, an article. The authors have granted ASM a non-exclusive, world-wide license to publish the Supplemental Material files. Please contact the corresponding author directly for reuse.
