# Supplementary material for: Intact, recombinant, and spliced forms of endogenous mouse mammary tumor viruses in inbred and wild mice
Source: J Virol. 2025 Mar 13;99(4):e00079-25. doi: 10.1128/jvi.00079-25 (PMC11998498; doi:10.1128/jvi.00079-25)
Supplement: Supplemental material — Figures S1 and S2, Tables S1 to S5, and Data Files S2 and S3. [file jvi.00079-25-s0002.pdf]

Supplementary Files for

Intact, recombinant and spliced forms of endogenous mouse mammary tumor viruses in inbred and wild mice

Oscar Lam[a,*], Esther Shaffer[a], Guney Boso[a,**], and Christine A. Kozak[a,#]

Supplementary Figures:

Figure S1. Sites under positive selection in the Tfrc receptor.

Figure S2. Phylogenetic trees of *sag* gene segments A, B and C defined in Figure 8.

Supplementary Tables:

Table S1. Repeats and rearrangements at *Mtv* insertion sites.

Table S2. Primer list.

Table S3. Insertion sites of Mtvs in regions of shared haplotype.

Table S4. Sources and trapping sites of wild mouse DNAs.

Table S5. Previously sequenced *Mtvs* and MMTV segments used for tree construction.

Supplementary Datafiles:

Datafile S1. Sequences of 28 *Mtvs* found in 17 sequenced mouse genomes. **Provided as separate supplementary text file.**

Datafile S2. Cellular sequences 3' to the *MtvBQC*.

Datafile S3. Alignment of the protein sequences of *Tfrc* exons 8 and 17 from wild mice relative to the sequence in the B6 reference genome.

Figure S1. Sites under positive selection in the *Mus Tfrc* receptor.

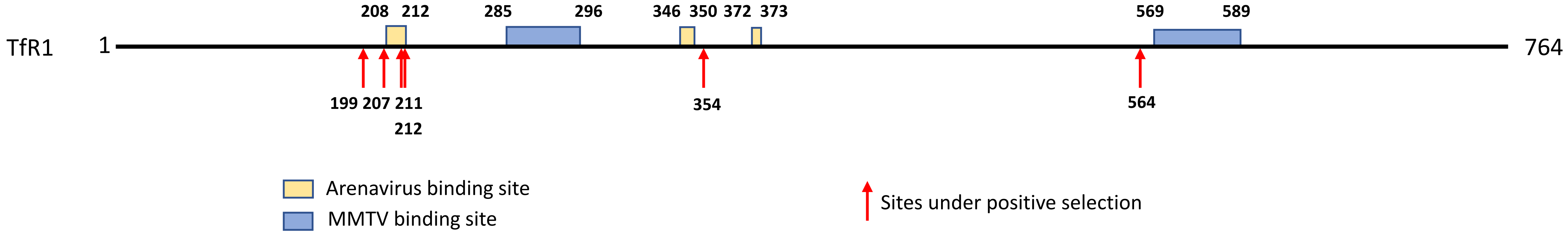

Figure S2. Phylogenetic trees of *sag* gene segments A, B and C defined in Figure 8. Maximum likelihood trees were generated using RaxML with aligned *sag* sequences from the indicated *Mtvs* for (A) GARD segment A (first 474 nucleotides), (B) GARD segment B (nucleotides 475-825) and (C) GARD segment C (nucleotides 826-end). Bootstrap values that are below 70 are shown at the relevant nodes. The tree was midrooted. The scale bar represents 0.02 or 0.05 nucleotide substitutions per site. Tips with accession numbers represent previously sequenced *Mtvs* (Table S4).

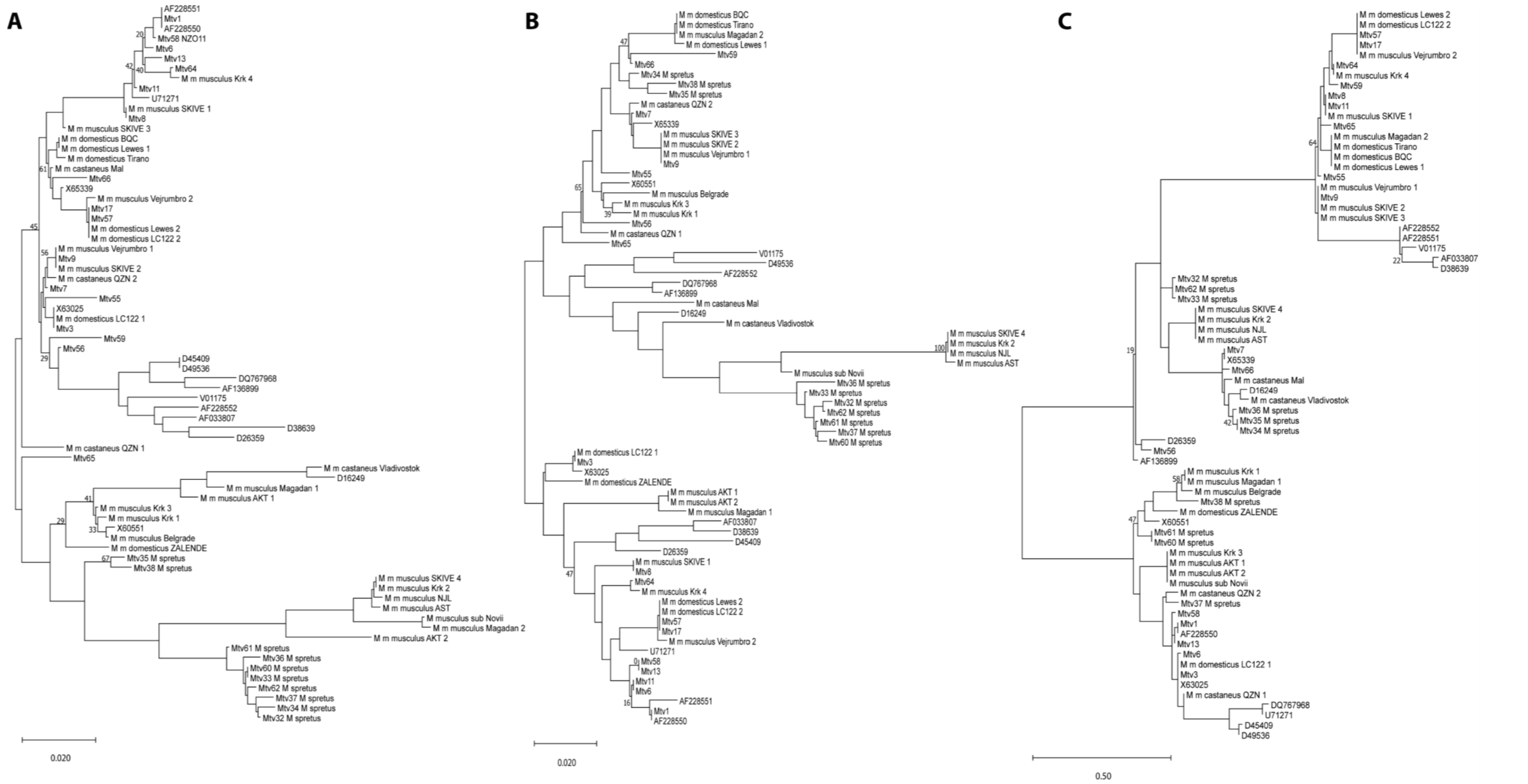

Table S1. Repeats and rearrangements at *Mtv* insertion sites.

**Sequence Type**	***Mtv***	**Mouse**	**Repeat at Insertion Site**	**Orthologous Site in B6 Reference Genome**
Simple repeats	13	FVB/NJ	GA (1263 bp)	Missing 900 bp
	38	SPRET/EiJ	TC (586 bp)	Missing
	66	WSB/EiJ	GA, AA (337 bp)	Missing 246 bp
Repetitive Elements	58	NZO/HlLtJ	GSAT_MM	
	59	LP/J	GSAT_MM	
Indels	11	C3H/HeJ		783 bp deletion spanning insertion
Duplications	3	NOD/ShiLtJ		Four linked copies of insertion site
	7	DBA/2J		Three linked copies of insertion site, segment translocated to Chr 14

Table S2. List of primers used for PCR

Mtv	5'flank	3'flank	Internal MMTV	Sizes in bp
Identification of individual Mtvs				
1	GGTTGAACTCCCGAGAGTGTC	GTGTCAATCTCTTCTCCACCTG	CCT TCA CTT TCC AGA GGG TC	
3	CTG TTC AAC CAC ACC TTG G	GCG GAG AGT CTG GTG AAA ATC C	GGT CCC CTA TAA GTC CCT GG	
6	CCA TGA TGC CAC ACA CAC G	GAG CAG TAC ATA GTG TGG AAC CCA C	GGT CCC CTA TAA GTC CCT GG	
7	GCT TCT GTC CAT TGG GTG G		GGT CCC CTA TAA GTC CCT GG	
7	CCA GGG TAC TGC TGA CTG TG		GGT CCC CTA TAA GTC CCT GG	
8	GCC ACG GTG ATC TGA TGA G	CAC AAA TGG AGG CAA AGC TGG	GGT CCC CTA TAA GTC CCT GG	
9	GAA TGC CAG GAC CAA GAA GTG G	CTC CTT CCA CTG ACT CCA GAC	CCT TCA CTT TCC AGA GGG TC	
11	CCT GTG GAT TGC TAC TGT GC	CCG AAG GTC CTA TCC CAT GAA CTC	GGT CCC CTA TAA GTC CCT GG	
13				
17	GTT AAG TGT GGG GGT GAC AG	GGA GGT CTC TCT GTC ATG TCC	GAG GAA GTT GGC TGT GGT C	
21	CAA CTG CTC TGC CCT ATC TTC	GGT AAT GGC TTC CAA TGA GG	GGT CCC CTA TAA GTC CCT GG	
55	CTG CCC ACT TTC TCC CTA C		GAC ACT CTC GGG AGT TCA ACC	
		GGT CAG GCA TGG TGA TTC CAC	CCT TCA CTT TCC AGA GGG TC	
56	GCA TGA GTG CCT TGT TTG CC	CAC CAG AAC CTG CCA GAG	GAC ACT CTC GGG AGT TCA ACC	
57	CAA GGT TTG GGT GGG CAT AG	GAT AAG CAG ATC CCT GGA GG	GGT CCC CTA TAA GTC CCT GG	
58	CTC GCC ATA TTC CAG GTC CTT C		GGT CCC CTA TAA GTC CCT GG	
59	GCC ATA TTC CAG GTC CTT CAG TG		GGT TGA ACT CCC GAG AGT GTC	
32		GGC AAA TCT GAA TCC CTC CCT G	CCA GAA TGT CCA CTA GGT GTC	
33	GGT GGT CTG TGG CAG TAT TTT CC	GAC TGG TCT GTG AGG CAT TC	GGC TCA CCG TAA CCT ACC TC	
34		CAC TCT GAG GCT GGG GTT AC	CCA GAA TGT CCA CTA GGT GTC	
34		CAA GCA GTA GGT GAC ATC CCT G	CCA GAA TGT CCA CTA GGT GTC	
35	CAC TGG TCT GAT GAC ACG GCT G		CCA GAA TGT CCA CTA GGT GTC	
35	GCT GAC AGA TAC ACC AGT GG		CCA GAA TGT CCA CTA GGT GTC	
36	CGA TGA AGG ATG GGG AAC TGG	GGA ACC TGC TGG TGA CAG AG	CCT TCA CTT TCC AGA GGG TC	
37	GTC TAT GAC CCC TCT GCT C	CTA ACC TCC CTC TTC ATC ATC TGG	CCA GAA TGT CCA CTA GGT GTC	
38	GCA GAC TGA AAC CTT TGC C	CAA CAA CAA CAG ATC CCA GCC	GGT CCC CTA TAA GTC CCT GG	
60	CCT CCA TTC TGG CAG CAC TTC C		ACA CCA AGG AGG TCT AGC	
61	GCA TGA CAG GAG TGA GTA CAC		ACA CCA AGG AGG TCT AGC	
62		CAA GTG TGA GTG CGG ACT ACA G	GGT TGA ACT CCC GAG AGT GTC	
63	GGA GAC CTT ACT AGG GTT GC		GAC ACT CTC GGG AGT TCA ACC	
63	CCT GGT TAC AGA AAA TGG CTG C		GAC CCT CTG GAA AGT GAA GG	
64	GCG AGA GAG GTT GTG TCA GC	CAT CAA GGC GTT CTG TTC GTG	GGT TGA ACT CCC GAG AGT GTC	
65	GTT CCT GGG AGG GAG AAT CTG	GGT AAC AAG GGG AGC AAG AG	GAG GAA GTT GGC TGT GGT C	
66	CAA CAC CCC ACT CTT ACC AGT G	CTT CCC TGA CCG AGT TTT C	GAC CCT CTG GAA AGT GAA GG	
Internal segments, *Mtvs* with internal deletions				
BQCMtv	GGT TGA ACT CCC GAG AGT GTC	GGC TCA CCG TAA CCT ACC TC		
	CCT TCA CTT TCC AGA GGG TC	CTT CTG CTC CTT GAC CTC C		
	GGA GGT CAA GGA GCA GAA G	CTA AAC CCG TGA AAG TCA GGC G		
	GAC CCT AGA CCC CAT CAA AG	GAC CCT CTG GAA AGT GAA GG		
SKIVEMtv	GGT TGA ACT CCC GAG AGT GTC	GAT AAG ACA CCA CAT CAC CCT G		
	CAG GGT GAT GTG GTG TCT TAT C	CTA AAC CCG TGA AAG TCA GGC G		
	GAC CCT AGA CCC CAT CAA AG	GAG GAA GTT GGC TGT GGT C		
ZALENDEMtv	GGT TGA ACT CCC GAG AGT GTC	CCT TCT GAG CCT CGT TGT TCC		

Table S3. Insertion sites of *Mtvs* in regions of shared haplotypes. Older *Mtvs* are always present in the same haplotype. For recent *Mtvs*, only some strains with the haplotype carry the *Mtv*.

Strains	Strain Groups	Recent *Mtvs*				Older *Mtvs*							
		1	3	13	23	6	7	8	9	11	14	17	21
C58/57	C57BL/6J	-	-	-	-	-		+	+	-	-	+	-
	C57BR/cdJ	-			-	-	-	+	+	+	-	+	-
	C58/J	-	+		-	-	+		-	-	-	+	-
	LT/SvEiJ	-			-		+	+	-	-	-	+	-
	C57L/J	-	-		-	-		+	+	+	-	+	-
Castle	1291/SvJ		-	+		-	-	+	+	+	-	+	
	A/J	-	-	+	+	+	-	+	-	-	-	-	-
	AKR/J	-	-	-	+	-	+	+	+	-	-	+	-
	BALB/cJ	-	NB	-	-	+	-	+	+	-	-	-	-
	C3H/HeJ	+	NB	-	-	+	-	+	-	+	+	-	-
	CBA/J	-	NB	-	-	+	+	+		-	+	+	-
	DBA/2J	+	NB	+	-	+	+	+	-	+	+	+	-
	I/LnJ	-	-		-	-	+	-	-	-	-	+	-
	LP/J	-	NB	+		+	-	+	+	+	-	+	-
	RF/J	+	-		+	-	+	+	-	-	-	+	-
	SEA/GnJ	-	NB		-	+	+	+	-	-	-	-	-
	SEC/1ReJ	-	NB		-	+	-	+	+	-	-	-	-
	SM/J	-	-		-	+	+	+	-	-	+	+	-
NZ	NZB/BINJ	-	+			-	+		+	-	+	+	
	NZO/HILtJ	-	+	-	-		+		-	-	+	+	+
	NZM2410/J					+	-	+		-			+
	NZW/LacJ		+		-		-	+	-	-		+	+
Swiss	FVB/NJ	-	-	+	-	-	-	+	-	-	+	+	-
	NOD/ShiLtJ	-	+	-	-	-	-		-	-	-	+	-
	NON/ShiLtJ	-	+		+	+	+	-		-	+	+	-
	NOR/LtJ	-	+		-	-	-		+	-	NB		-
	SJL/J		-		-	-	-	+	-	-	-	-	-
	SWR/J	-	-		-	-	+	+	-	-	+	+	-
Other	TALLYHO/JngJ	-	NB		-	-	-	+	-	-	+	+	-
	KK/HIJ		+		-	+	-		-	-	-		-
	LG/J	-	-		-	-	-	+	+	-	+	+	-
	PL/J	-	+		+	-	-	+	-	-	-	+	-
	RIIIS/J	-	-		-	+	-	+	-	-	+	-	-

Gray blocks: strains in which the *Mtv* is present within genomic haplotype segments of at least 90kb defined by identical SNPs.
Orange blocks: strains in which the *Mtv* is not present in the expected haplotype segment.
Black blocks: strains with genomic substitutions from *M. m. castaneus* or *M. m. musculus* at the integration site.
NB: no band.

Table S4. Sources of wild-caught and wild-derived mice and mouse DNAs.

Mus species	Mus musculus subspecies[1]	Alternate designation	Trapping location	DNA Code	Source	Used for	
						Mtv typing	Trees
musculus	*bactrianus*	M.Bac	Mashhad, Iran	MG-5047	RIKEN	X	
	castaneus	Bal	Bandar,Sumatra, Indonesia	MG-5121	RIKEN	X	
	castaneus	CAS/Li	Thonburi, Thailand		Potter	X	
	castaneus	CASA/RkJ	Thailand		Jackson	X	
	castaneus	CASP/1Nga	Los Banos, Philippines		RIKEN	X	
	castaneus	CAST/17	Pathum Thani, Thailand		Hartley	X	
	castaneus	CAST/EiJ	Thonburi, Thailand		Jackson	X	*Trf1*
	castaneus	CAST/N	Thonburi, Thailand		Potter	X	*Trf1*
	castaneus	CAST/Rp	Thonburi, Thailand		Roswell Park	X	
	castaneus	HMI/Ms	Hemei, Taiwan		RIKEN, Abe	X	
	castaneus	Mal	Pahang, Malaysia	MG-5060	RIKEN	X	*env, pol, sag*
	castaneus	MYS/Mz			RIKEN	X	
	castaneus	Nin	Ningpo, Zhejiang, China	MG-0795	RIKEN	X	
	castaneus	Qzn	Quezon City, Philippines	MG-0421	RIKEN	X	*pol, sag*
	castaneus		Vladivostok, Russia	MG-3077	RIKEN	X	*sag*
	domesticus	ABUR	Abu Rawash, Egypt		Potter	X	*env*
	domesticus	BIBB	Sede Boker, Israel	MG-0377	RIKEN	X	
	domesticus	BQC	Bouquet Canyon, CA		Potter	X	*pol, env, sag, Trf1*
	domesticus	CALB/RkJ	California		Jackson	X	
	domesticus	CalWM (7 mice)	Lake Casitas, CA		Rasheed	X	*env, sag, Trf1*
	domesticus	CLA (Centreville Lite)	Centreville, MD		Potter	X	
	domesticus	DFC	Ucciani, Corsica, France	MG-0385	RIKEN	X	
	domesticus	HAF (Havens Farm)	Davidsonville, MD		Potter	X	*Trf1*
	domesticus	JJD (J.J.Downs)	Ridgely, MD		Potter	X	*env*
	domesticus	LW, LEWES/EiJ	Lewes, DE		Potter, Jackson	X	*env, sag*
	domesticus	PERC/EiJ	Rimac Valley, Peru		Jackson	X	

	domesticus	PGN2	Pegion region, Canada		Abe	X	
	domesticus	PRAE, praetextus	Erfoud, Morocco		Potter	X	*Trf1*
	domesticus	SAF (Sanners Farm)	Davidsonville, MD		Potter	X	
	domesticus	SC-1 cells	California		Hartley	X	
	domesticus	SF/CamEiJ	Marin County, CA		Jackson	X	
	domesticus	SK/Cam	Skokholm Is., U.K.	MG-0067	RIKEN	X	
	domesticus	TIRANO, poschiavinus	Tirano, Italy		Potter	X	*env, sag, Trf1*
	domesticus	ZALENDE, poschiavinus	Zalende, Switzerland		Potter	X	*env, sag, Trf1*
	domesticus	WMP/PasDnJ	Monastir, Tunisia		Jackson	X	
	domesticus	WSB (Watkins Star)	Watkins Farm, MD		Potter	X	*Trf1*
	molossinus	Aiz1	Aizuwakamatsu, Fukushima, Japan	MG-0489	RIKEN	X	
	molossinus	Hkz	Hakozaki, Fukuoka, Japan	MG-0461	RIKEN	X	
	molossinus	JF1	Fancy mouse stock		Jackson	X	
	molossinus	Kgs	Kagoshima, Kagoshima, Japan	MG-5013	RIKEN	X	
	molossinus	Kor	Koriyama, Fukushima, Japan	MG-0492	RIKEN	X	*Trf1*
	molossinus	Mol/Li	Kyushu, Japan		Potter	X	
	molossinus	MOLD/RkJ, MOLF/EiJ, MOLG/DnJ, MOLC/Rk	Fukuoka, Kyushu, Japan		Jackson	X	*Trf1*
	molossinus	Mro	Morioka, Iwate, Japan	MG-0271	RIKEN	X	
	molossinus	MSM	Mishima, Shizuoka, Japan	MG-0082	RIKEN, Abe	X	
	molossinus	MZH (MOM)	Mizuho, Aichi, Japan	MG-0099	RIKEN	X	
	molossinus	Nig	Niigata, Honshu, Japan	MG-0202	RIKEN	X	
	molossinus	Ohm	Oma, Aomori, Japan	MG-0282	RIKEN	X	
	molossinus		Higashi-Ohmiya, Saitama, Japan	MG-0210	RIKEN	X	
	molossinus		Nirayama, Shizuoka, Japan	MG-0219	RIKEN	X	

	molossinus		Mito, Ibaraki, Japan	MG-0235	RIKEN	X	
	molossinus		Takatsuki, Osaka, Japan	MG-0240	RIKEN	X	
	molossinus		Kanazawa, Ishikawa, Japan	MG-0255	RIKEN	X	
	molossinus		Ashiro, Iwate, Japan	MG-0257	RIKEN	X	
	molossinus		Ohmiya, Saitama, Japan	MG-0335	RIKEN	X	
	molossinus		Kyoto, Japan	MG-0422	RIKEN	X	
	molossinus		Shizuoka, Shizuoka, Japan	MG-0427	RIKEN	X	
	molossinus		Tanegashima, Kagoshima, Japan	MG-0442	RIKEN	X	
	musculus	Akt/TUA	Aktyubinsk, Kazakhstan		RIKEN	X	*env, sag*
	musculus	Ast/TUA (wagneri)	Astrakhan, Russia		RIKEN	X	*sag*
	musculus	BLG2	Toshevo, Bulgaria		Abe	X	
	musculus	CZI, CZECH/EiJ	Morovia, Czech Republic		Potter	X	*env, Trf1*
	musculus	CzII, CZECHII/EiJ	Bratislava, Slovakia		Potter	X	*Trf1*
	musculus	GOR/TUA	Gorno-Altaisk, Russia		RIKEN	X	
	musculus	IRK/TUA	Irkutsk, E. Siberia, Russia		RIKEN	X	
	musculus	Jix	Tianjin, China	MG-0843	RIKEN	X	
	musculus	KAZ/TUA	Alma-Ata, Kazakhstan		RIKEN	X	
	musculus	Krk1	Krakow, Poland	MG-5235	RIKEN	X	*env, sag*
	musculus	MBT/Pas	Toshevo, Bulgaria		RIKEN	X	
	musculus	MYL	Ljubljana Slovenia	MG-0399	RIKEN	X	*env*
	musculus	NJL	Northern Jutland, Denmark		Abe, RIKEN	X	*sag*
	musculus	PWD/PhJ	Kunratice, Czech Republic		Jackson	X	
	musculus	PWK/PhJ	Lhotka, Czech Republic		Jackson	X	
	musculus	Skive	Skive, Denmark		Potter	X	*env, sag, Trf1*
	musculus	Tom/TUA	Tomsk,Tuva, Siberia, Russia		RIKEN	X	
	musculus	VEJ	Vejrumbro, Denmark		Potter, Morse	X	*env, sag*
	musculus		Belgrade, Serbia		Morse	X	*env, sag*

	musculus		Brno, Czech Republic		Morse	X	
	musculus		Viborg, Denmark		Morse	X	*pol*
	musculus (gansuensis)	Aks	Aksu, Xinjiang, China	MG-0608	RIKEN	X	*pol, env*
	musculus (gansuensis)	Htn	Hotan, Xinjiang, China	MG-0762	RIKEN	X	
	musculus (gansuensis)	M.sub-Kes	Kashi, Xinjiang, China	MG-0686	RIKEN	X	
	musculus (homourus)	M.sub-Jin	Jinan, Shandong, China	MG-0784	RIKEN	X	
	musculus (tantillus)	Las	Lasa, China	MG-0723	RIKEN	X	
	musculus (wagneri)	KNB/TUA	Balkhash Lake, Kazakhstan		RIKEN	X	
	musculus (wagneri)	wag-ton	Tongliao, Inner Mongolia	MG-0856	RIKEN		
	spp.	Bjn2	Dongcheng, Beijing, China	MG-5066	RIKEN	X	*Trf1*
	spp.	Dal	Dali, Yunnan, China	MG-0788	RIKEN	X	
	spp.	Gui	Guilin, Guangxi, China	MG-0501	RIKEN	X	
	spp.	Gui	Guilin, Guangxi, China	MG-0502	RIKEN	X	
		Ias3	Suwoni, Gyeonggi-do, Korea	MG-5018	RIKEN		
	spp.	Jia	Jiangyin, Uuxi, Jiangsu, China	MG-2100	RIKEN	X	
	spp.	Jin	Jinan, Shandong, China	MG-0790	RIKEN	X	
	spp.	Kjr	Kojuri, Korea	MG-0060	RIKEN	X	
	spp.	Kun	Kunming, Yunnan, China	MG-0529	RIKEN	X	
	spp.	Lai	Laiyang, Yantai, Shandong, China	MG-2022	RIKEN	X	
	spp.	Las	Lasa, China	MG-0721	RIKEN	X	
	spp.	Lzh	Lanzhou, Gansu, China	MG-0507	RIKEN	X	
	spp.	Moh	Mohe, Buryatia, China	MG-0670	RIKEN	X	*env*
	spp.	Novii	Novii settlement, Maysky, Russia	MG-3012	RIKEN	X	*sag*
	spp.	Qiq	Qiqihar, Heilongjiang, China	MG-0993	RIKEN	X	
	spp.	Qiq	Qiqihare, Heilongjiang, China	MG-0992	RIKEN	X	

	spp.	Tac	Tacheng ,China	MG-0611	RIKEN	X	
	spp.	Wuh	Wuhan, Hubei, China	MG-0908	RIKEN	X	
	spp.	Wuh	Wuhan, Hubei, China	MG-0950	RIKEN	X	
	spp.	Yaz	Yangzhou, China	MG-0715	RIKEN	X	
	spp.	Zhj	Zhenjiang, Jiangsu, China	MG-0713	RIKEN	X	
	spp.	Zhj	Zhenjiang, Jiangsu, China	MG-0714	RIKEN	X	
	spp.		Birakan Settlement, Khabarovski region, Western Birobidzan city, Russia	MG-3027	RIKEN	X	
	spp.		Blagovetsensk, Amur, Russia	MG-3064	RIKEN	X	
	spp.		Busan, Korea	MG-0444	RIKEN	X	
	spp.		Busan, Korea	MG-0445	RIKEN	X	*Trf1*
	spp.		Donetsk, Ukraine	MG-3065	RIKEN	X	*env*
	spp.		Grozny City, Chechnya, Russia	MG-3010	RIKEN	X	
		Guz(Shi)	Guangzhou, Guangdang, China	MG-0503	RIKEN		
	spp.		Innokentevka, Buryatia, Russia	MG-3073	RIKEN	X	
	spp.		Magadan, Russia	MG-3063	RIKEN	X	*env, pol, sag*
	spp.		Chevnogolvka, Moscow, Russia	MG-3056	RIKEN	X	*env*
	spp.		Moscow, Russia	MG-3058	RIKEN	X	
	spp.		Novosibirsk, Russia	MG-3054	RIKEN	X	*env*
	spp.		Teli settlement, Russia	MG-3004	RIKEN	X	
	spp.		Vladivostok, Russia	MG-3034	RIKEN	X	
	spp.		Vladivostok, Russia	MG-3025	RIKEN	X	
	spp.		Yu-Sahalinsk, Russia	MG-3047	RIKEN	X	
caroli			Chonburi, Thailand		Potter		
cervicolor			Thailand		Potter		
famulus			India		Hartley		
fragilicauda			Thailand		Hartley		
macedonicus			Bulgaria		Elliott		

pahari			Tak Province, Thailand		Potter		
platythrix			India		Potter		
setulosus					Potter		
spicilegus			Halbturn, Austria		Potter		
spretus			Puerto Real, Spain		Potter		
terricolor			India		Eiden		

[1]Samples sorted according to genetic variation/relatedness and trapping site. spp., species undetermined

Table S5. Previously sequenced MMTV XRVs and ERVs.

GenBank Accession No.	Name	Mouse Source	Use
AF033807			*sag,env,pol* trees
AF043690		Cell line T-25-Adh	*env* tree
AF071010		RIII	*env* tree
AF136899	RIII/Sa-MMTV-2	RIII/Sa	*sag* tree
AF228550	Mtv1	C3H	*sag,env,pol* trees
AF228551	HeJ	C3H	*sag,env* tree
AF228552	C3H	C3H	*sag,env,pol* trees
D16249	JYG		*sag,pol,env* trees
D26359	MMTVBSA	Japanese fancy mouse FM	*sag* tree
D38639	II Tes	DBA x Japanese mice	*sag* tree
D45409	II Tes2		*sag* tree
D49536	Mtv51	Japanese pet mouse X SII	*sag* tree
DQ223969	rem		*env* tree
DQ767968	RIII/Sa-1 MMTV	RIII/Sa	*sag,env* trees
K00556	MMTV env	C3H	*env* tree
U71271	BALB2MMTV LA	BALB/cT	*sag* tree
V01175		GR	*sag* tree
X60551	MTV-MAI	MAI	*sag* tree
X63025		C58	*sag* tree
X64554	Mtv6 sag	DBA/2	*sag* tree
X64555	Mtv13 sag	DBA/2	*sag* tree
X65339	MMTV(SW)	BALB	*sag* tree

Datafile S2. Cellular sequences 3’ to *BQCMtv.* The sequence was identified as the major satellite, GSAT-MM by Dfam. The 3’ end of the provirus is in lower case.

cctcaggtcggccgactgcggcagGCAAGAAAACTGAAAATCACGGAAAATGAGAAATACACACTTTAGGACGTGAAATATGGCGAGGAAAACTGAAAAAGGTGGAAAAT
TTAGAAATGTCCACTGTAGGACGTGGAATATGGCAAGAAAACTGAAAATCATGGAAAATGAGAAACATCCACTTGACGACTTGAAAAATGACGAAATCACTAAAAAA
CGTGAAAAATGAGAAATGCACACTGAAGGACCTGGAATAAGGCGAGAAAACTGAAAATCACGGAAAATGAGAAATACACACTTT

Datafile S3. Alignment of the protein sequences of *Tfrc* exons 8 and 17 from wild mice relative to the sequence in the B6 reference genome. Regions implicated in virus binding are highlighted, with key residues in red.

Exon 8

```
BC054522.1                  VIVRAGEITFAEKVANAQSFNAIGVLIYMDKNKFPVVEADLALFGHAHLGTG
M.m.castaneus-CAST/N        ............-.......................................
M.m.molossinus-Fukushima    ....................................................
M.m.musculus-SKIVE          ....................................................
M.m.musculus-BJN2           ....................................................
M.m.musculus-Pusan          ....................................................
M.m.musculus_CZII           ....................................................
M.m.domesticus-BQC          ....................................................
M.m.domesticus-TIRANO       ....................................................
M.m.domesticus-ZALENDE      ....................................................
M.m.domesticus-LC107        ....................................................
```

Exon 17

```
BC054522.1                  DADYPYLGTRLDTYEALTQKVPQLNQMVRTAAEVAGQLIIKLTHDVELNLDYEMYNSKLLSFMKDLNQFKTDIR
M.m.castaneus-CAST/N        ..........................................................................
M.m.molossinus-Fukushima    ..........................................................................
M.m.musculus-SKIVE          ..........................................................................
M.m.musculus-BJN2           ..........................................................................
M.m.musculus-Pusan          ..........................................................................
M.m.musculus_CZII           ..........................................................................
M.m.domesticus-BQC          ..........................................................................
M.m.domesticus-TIRANO       ..........................................................................
M.m.domesticus-ZALENDE      ..........................................................................
M.m.domesticus-LC107        ..........................................................................
```
